# Human blastoids model blastocyst development and implantation

**DOI:** 10.1038/s41586-021-04267-8

**Published:** 2021-12-02

**Authors:** Harunobu Kagawa, Alok Javali, Heidar Heidari Khoei, Theresa Maria Sommer, Giovanni Sestini, Maria Novatchkova, Yvonne Scholte op Reimer, Gaël Castel, Alexandre Bruneau, Nina Maenhoudt, Jenna Lammers, Sophie Loubersac, Thomas Freour, Hugo Vankelecom, Laurent David, Nicolas Rivron

**Affiliations:** 1 Institute of Molecular Biotechnology of the Austrian Academy of Sciences (IMBA), Vienna BioCenter (VBC), Vienna, Austria; 2 Institute of Molecular Pathology (IMP), Vienna Biocenter, Vienna, Austria; 3 Université de Nantes, CHU Nantes, INSERM, Centre de Recherche en Transplantation et Immunologie, UMR 1064, ITUN, Nantes, France; 4 Unit of Stem Cell Research, Cluster of Stem Cell and Developmental Biology, Department of Development and Regeneration, KU Leuven, (University of Leuven), Leuven, Belgium; 5 CHU Nantes, Service de Biologie de la Reproduction, Nantes, France; 6 Université de Nantes, CHU Nantes, INSERM, CNRS, SFR Santé, FED 4203, INSERM UMS 016, CNRS UMS 3556, Nantes, France

**Keywords:** Embryonic induction, Embryonic stem cells

## Abstract

One week after fertilization, human embryos implant into the uterus. This event requires the embryo to form a blastocyst consisting of a sphere encircling a cavity lodging the embryo proper. Stem cells can form a blastocyst model that we called a blastoid^1^. Here we show that naive human pluripotent stem cells cultured in PXGL medium^2^ and triply inhibited for the Hippo, TGF-β and ERK pathways efficiently (with more than 70% efficiency) form blastoids generating blastocyst-stage analogues of the three founding lineages (more than 97% trophectoderm, epiblast and primitive endoderm) according to the sequence and timing of blastocyst development. Blastoids spontaneously form the first axis, and we observe that the epiblast induces the local maturation of the polar trophectoderm, thereby endowing blastoids with the capacity to directionally attach to hormonally stimulated endometrial cells, as during implantation. Thus, we propose that such a human blastoid is a faithful, scalable and ethical model for investigating human implantation and development^3,4^.

## Abstract (web summary)

Blastoids derived from naive PXGL-cultured human pluripotent stem cells in which Hippo, TGF-β and ERK pathways are inhibited closely recapitulate aspects of blastocyst development, form cells resembling blastocyst-stage cells and thus provide a model system for implantation and development studies.

## Main

A model of the human blastocyst would support scientific and medical progress. Its ability to predict human development will, however, depend on its ability to reproduce the sequences of blastocyst cellular determination and morphogenesis effectively, faithfully, and according to the developmental sequence and pace. Such modelling would ensure the formation of cells that reflect the blastocyst stage as a starting point to recapitulate aspects of subsequent developmental steps, including implantation. During this year, diverse ways of forming models of the human blastocyst have been proposed^5–9^. However, the cells generated often do not match those of the blastocyst^5,7–9^ (at 5–7 days post fertilization (dpf)) and have been proposed to rather reflect later developmental stages, including gastrulation (E14) and germ layers (mesoderm and endoderm) stages^10^. Here we form a model of the human blastocyst that specifically generates and spatially patterns cellular analogues of the blastocyst stage with similar developmental sequence and pace, which enables the model to mimic aspects of implantation.

## Inhibition of Hippo, ERK and TGFβ pathways

**Fig. 1:**
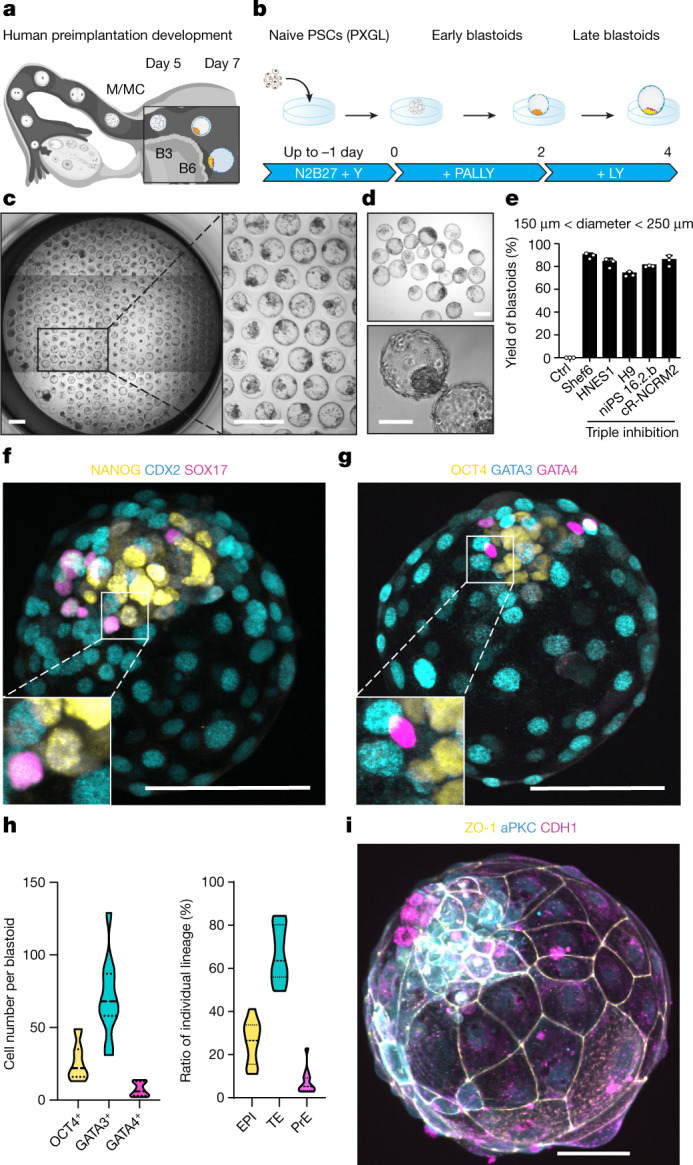
Triply inhibited naive PSCs efficiently form human blastocyst-like structures comprising analogues of the three founding lineages. **a**, A schematic of the time window of human peri-implantation development modelled by blastoids (days 5–7). M/MC, morula/morula compacted; B, blastocyst. **b**, One-step protocol of human blastocyst-like structure formation. N2B27, serum-free medium; PALLY, PD0325901 + A83-01 + LPA + hLIF + Y-27632. **c**, Phase-contrast image of human blastocyst-like structures formed on a non-adherent hydrogel microwell array after 96 h. Each microwell is 200 μm in diameter. Scale bars, 400 μm. **d**, Phase-contrast images of representative human blastocyst-like structures harvested from microwells. Scale bars, 200 μm (top) and 100 μm (bottom). **e**, Percentages of microwells including a human blastocyst-like structure for different naive PSC lines cultured in the PALLY condition with optimized LPA concentration compared with a H9 control (Ctrl) deprived of the three inhibitors. The morphometric definition of blastocyst-like structures is provided in Methods. *n* = 3 microwell arrays; mean ± s.d. **f**, **g**, Immunofluorescence of the epiblast (EPI) markers NANOG (yellow) (**f**) and OCT4 (yellow) (**g**), the TE markers CDX2 (cyan) (**f**) and GATA3 (cyan) (**g**), and the PrE markers SOX17 (magenta) (**f**) and GATA4 (magenta) (**g**) in human blastocyst-like structures. Scale bars, 100 μm. **h**, Absolute number of cells positive for OCT4, GATA3 and GATA4 (left) and ratios of cells belonging to individual lineages represented as percentage of total number of cells (right) in blastocyst-like structures (96 h) based on immunofluorescence. **i**, Representative immunofluorescence of the tight junction molecule ZO-1 (yellow), the adherence junction molecule CDH1 (magenta) and the apical domain molecule aPKC (cyan) in a representative human blastocyst-like structure. Scale bar, 50 μm. Source data

**Extended Data Fig. 1:**
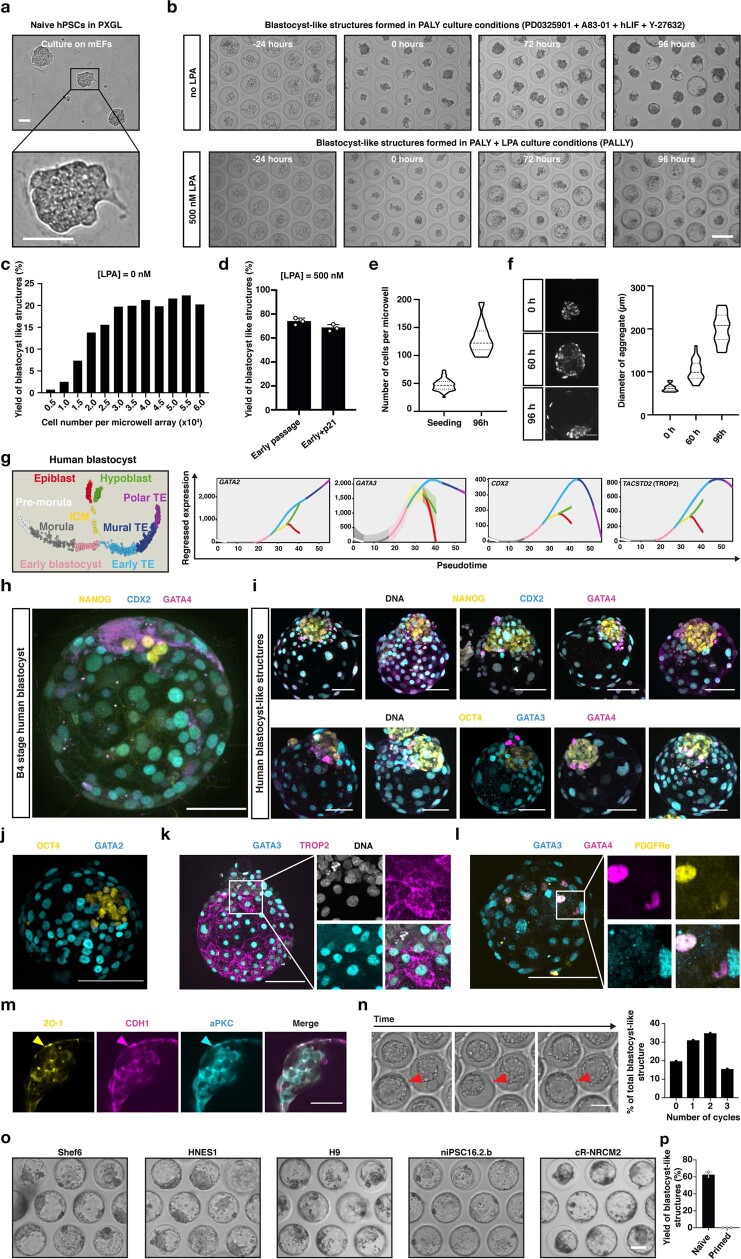
Naive hPSCs form human blastocyst-like structures comprising analogs of the three founding lineages. **a**. Phase contrast images of naive hPSCs cultured in PXGL medium and on MEF feeder layers. Scale bar: 50 μm. **b**. Time course phase contrast images of naive hPSCs aggregates cultured within microwell arrays either without LPA (PALY medium, **top**) or with 500 nM LPA (PALLY medium, **bottom**). Scale bar: 200 μm. **c**. Quantification of the effect of the initial cell numbers per microwell array on the yield of blastocyst-like structures. n=1 microwell arrays. **d**. Quantification of the effect of serial passaging of naive hPSCs on the yield of blastocyst-like structures. n=3 microwell arrays. mean± S.D. **e**. Quantification of the cell numbers per microwell at the time of seeding and in blastocyst-like structures at 96 h when cells are seeded at 3.0 x 10^4^ cells per microwell array. n=190 microwells (seeding) and n=12 blastocyst-like structures (96 hrs.). **f**. Fluorescence staining of DNA using Hoechst in representative naive hPSCs aggregates over the course of formation of blastocyst-like structures (96 h, **left**). Scale bar: 50 μm. Measurement of the distributed diameters of the structures over the course of formation of blastocyst-like structures (**right**). n=15, 31 and 11 for 0, 60 and 96 h, respectively. **g**. Pseudotime analysis of human pre-implantation development showing the expression of the TE markers *GATA2, GATA3, CDX2 and TACSTD2*. Gene expression analysis was performed by using the public data analysis tool (https://bird2cluster.univ-nantes.fr/demo/PseudoTimeUI/). **h**. Immunofluorescence stainings for EPI marker NANOG (Yellow), TE marker CDX2 (Cyan) and primitive endoderm marker GATA4 (Magenta) in a representative B4-stage human blastocyst. Scale bar: 50 μm. **i**. Immunofluorescence stainings for the EPI markers (Yellow) NANOG (**top**) and OCT4 (**bottom**); the TE markers (Cyan) CDX2 (**top**) and GATA3 (**bottom**); and the primitive endoderm marker (Magenta) GATA4 in five representative blastocyst-like structures. Counterstain with Hoechst (Grey) marking DNA. Scale bar: 50 μm. **j**. Immunofluorescence staining for EPI marker OCT4 (yellow) and TE marker GATA2 (Cyan) in blastocyst-like structures. Scale bar: 100 μm. **k**. Immunofluorescence staining for TE markers GATA3 (Cyan) and TROP2 (Magenta) in blastocyst-like structures. Scale bar: 100 μm. **l**. Immunofluorescence staining for TE markers GATA3 (Cyan) and PrE marker GATA4 (Magenta) and PDGFRa (Yellow) in blastocyst-like structures. Scale bar: 100 μm. **m**. Single optical section of immunofluorescence staining image for the tight junction molecule ZO-1 (Yellow), the adherence junction molecule CDH1 (Magenta), and the apical domain molecule aPKC (Cyan) in a representative human blastocyst-like structures. Scale bars: 50 μm. **n**. Representative time points from a timelapse image of naive cell aggregates, cavitating into blastocyst-like structures while showing cycles of cavity inflation and deflation (**left**) - quantification of blastocyst-like structures showing distinct frequencies of inflation and deflation (**right**). n=1 microwell arrays. Scale bar: 100 μm. **o**. Phase contrast images of representative areas of microwell arrays showing blastocyst-like structures formed from different naive hPSCs and hiPSCs lines. n>3. Scale bar: 100 μm. **p**. Quantification of the yield of blastocyst-like structures obtained from naive and primed H9 hPSCs. n=3 microwell arrays. mean± S.D. Source data

At 4 dpf, the conceptus forms a morula that initiates cavitation to make a blastocyst. Blastocyst development (at 5–7 dpf) supports the generation of the three founding lineages^11^: the epiblast (EPI), which is embryonic; trophectoderm (TE), which is extraembryonic; and primitive endoderm (PrE), which is extraembryonic (Fig. 1a). Peripheral cells become TE through inhibition of the Hippo pathway^12,13^. Naive human pluripotent stem cells (PSCs) cultured in PXGL^2^ efficiently form TE analogues upon inhibition of TGFβ and ERK pathways^14–16^. We aggregated naive PSCs in non-adherent hydrogel microwells and inhibited these three pathways (Fig. 1b, Extended Data Fig. 1ac). Upon exposure to lysophosphatidic acid (LPA) (a Hippo pathway inhibitor), A83-01 (an inhibitor of TGFβ family receptors) and PD0325901 (an ERK inhibitor) in a chemically defined medium containing the STAT activator leukaemia inhibitory factor (LIF) and Y-27632 (a ROCK inhibitor), blastocyst-like structures formed efficiently (Fig. 1c–e, Supplementary Videos 1, 2; more than 70% efficiency, diameters 150–250 μm; full morphometric criteria are presented in Methods) and consistently (Extended Data Fig. 1d, more than 20 passages). LPA was essential for this high efficiency (Extended Data Fig. 1b–d). Within 4 days, the cell number (47 ± 9 to 129 ± 27) and overall size (65–200 µm) had increased (Extended Data Fig. 1e, f) to ranges similar to those for 5–7 dpf blastocysts^17^ (stages B3 to B6). TE cell analogues^11^ (identified as GATA2^+^GATA3^+^CDX2^+^TROP2^+^) formed, proliferated (Fig. 1f–h, Extended Data Fig. 1g–l), and established adherens junctions (marked by epithelial cadherin (CDH1)), apical–basal polarity (indicated by atypical PKC (aPKC) localization) and tight junctions (marked by ZO-1; Fig. 1i, Extended Data Fig. 1m) while undergoing cycles of inflations and deflations^18^ (Extended Data Fig. 1n, Supplementary Video 2). Of note, all blastocyst-like structures set apart a unique inner cell cluster reflecting the EPI (OCT4^+^; 27 ± 13 cells; 26% of total cells) and PrE (GATA4^+^SOX17^+^PDGFRa^+^; 7 ± 5 cells; 7% of total cells) (Fig. 1f–h, Extended Data Fig. 1i, j, l). Multiple lines of naive human embryonic stem (ES) cells (Shef6, H9 and HNES1) and human naive induced PSCs (niPSC 16.2.b and cR-NCRM2) formed similar structures with comparably high efficiency (Fig. 1e, Extended Data Fig. 1o), whereas primed PSCs that reflect the post-implantation EPI did not (Extended Data Fig. 1p).

## Formation of blastocyst-stage analogues

**Fig. 2:**
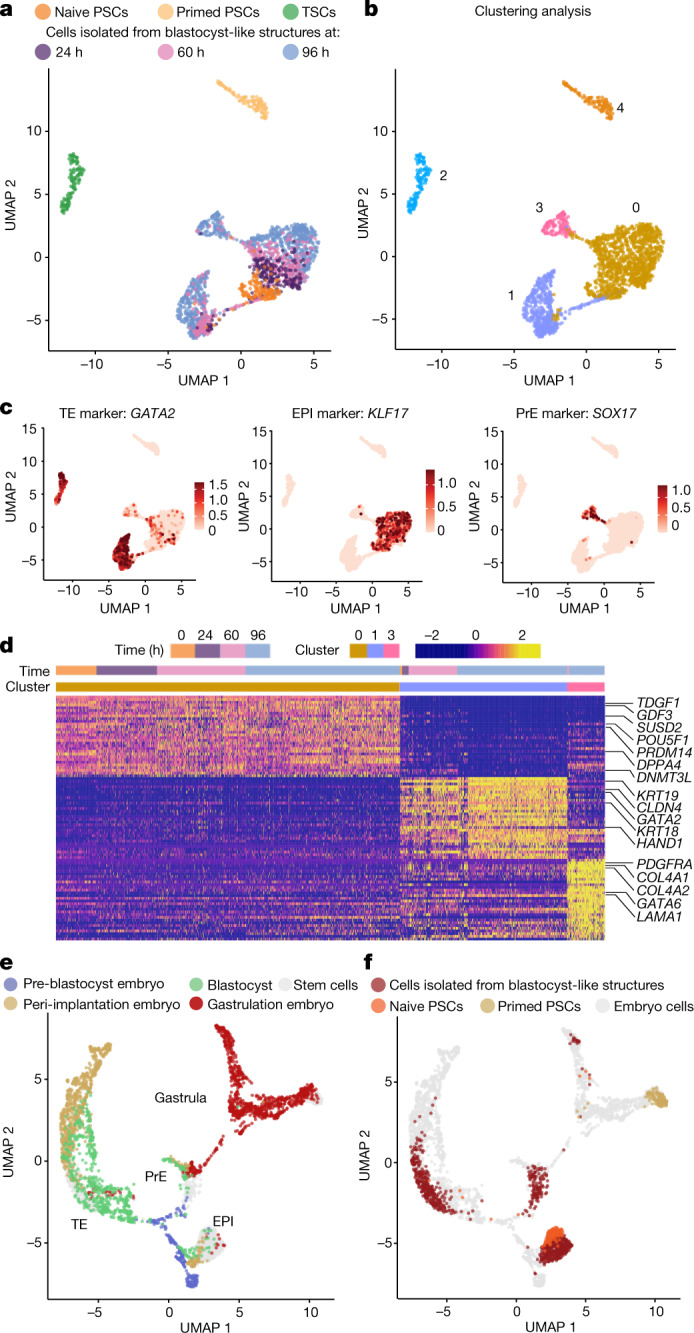
Human blastocyst-like structures form analogues of the three pre-implantation lineages. **a**, **b**, Uniform manifold approximation and projection (UMAP) of the transcriptome of single cells originating from blastocyst-like structures (at 24, 60 and 96 h), naive PSCs, primed PSCs and TSCs (representing post-implantation cytotrophoblasts); individual cells are coloured on the basis of their origin (**a**) or their unsupervised cluster affiliation (**b**). **c**, Expression level of markers of each blastocyst lineage. **d**, Unsupervised distance map generated using the top 30 genes that are enriched in clusters 0, 1 and 3 (defined in the UMAP in **b**). Note that this list includes epiblast markers specific to the blastocyst stage (for example, SUSD2, KLF17 and PRDM14). **e**, **f**, UMAP of single-cell transcriptome of cells from blastocyst-like structures, naive PSCs and primed PSCs integrated with published datasets from human embryos at pre-implantation, peri-implantation (in vitro cultured blastocysts) and gastrulation (Carnegie stage 7, that is, between embryonic days 16 and 19) stages. Individual cells are coloured on the basis of their origin in human embryos (**e**) or blastocyst-like structures or stem cells (**f**).

**Extended Data Fig. 2:**
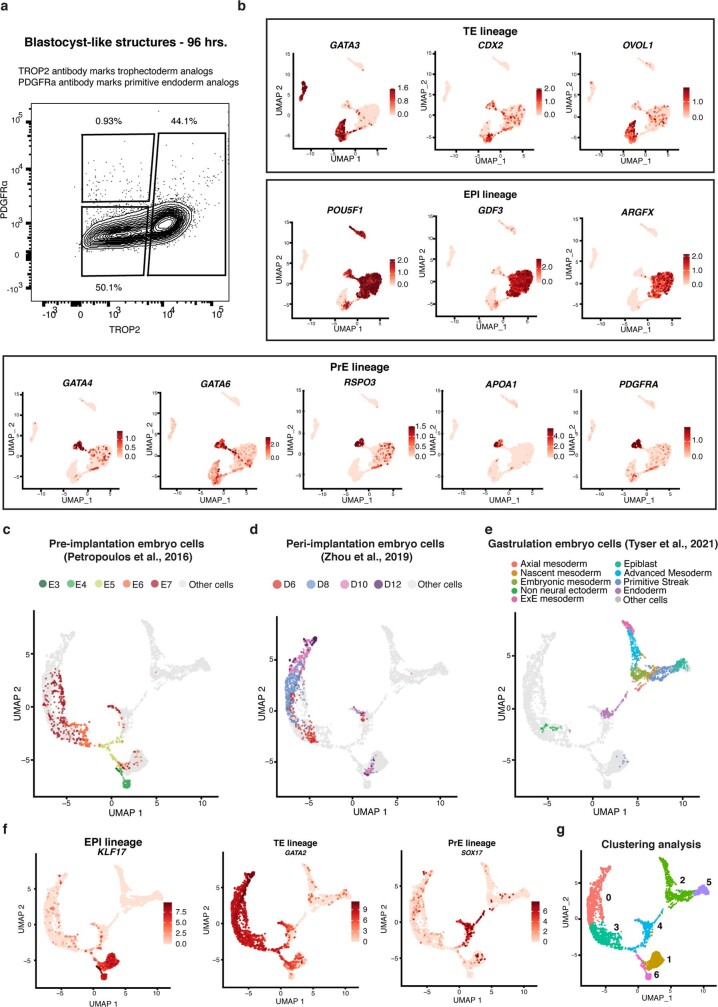
Human blastocyst-like structures form analogs of pre-implantation lineages. **a**. Flow cytometry analysis plot of cells isolated from blastocyst-like structures and stained for lineage-specific surface markers PDGFRa (PrE) and TROP2 (TE). The gates were used to sort analogs of EPI (double negative), TE (TROP2^high^) and PrE (PDGFRα^high^) to subsequently process for single cell RNA sequencing. Note that the gates did not exclude any cells. This analysis was performed to correlate RNA measures, while ensuring a representation of all cell types. **b**. UMAPs of the transcriptome of single cells isolated from blastocyst-like structures and displaying the expression levels of genes specific for each of the three blastocyst lineages (TE - Trophectoderm, EPI - Epiblast, and PrE- Primitive endoderm). **c-g**. UMAPs of single cells isolated from both blastocyst-like structures and from embryos ranging from E3 to E19. **c**. Coloration of cells originating from *In Vitro Fertilization* (IVF) embryos isolated on day 3 (E3) to day 7 (E7). This period comprises only pre-implantation stage embryos. **d**. Coloration of cells originating from IVF embryos isolated on day 6 (E6) to day 12 (E12). These blastocysts (E6) were cultured *in vitro*. Note that this annotation reflects the number of days in culture rather than the developmental stages. **e**. Coloration of cells originating from gastrulation-stage embryo isolated on day 17 (E17) to 19 (E19). **f**. The expression levels of genes specific for each of the three blastocyst lineages (EPI, TE, and PrE). **g**. Coloration of cells displaying their unsupervised cluster affiliation.

**Extended Data Fig. 3:**
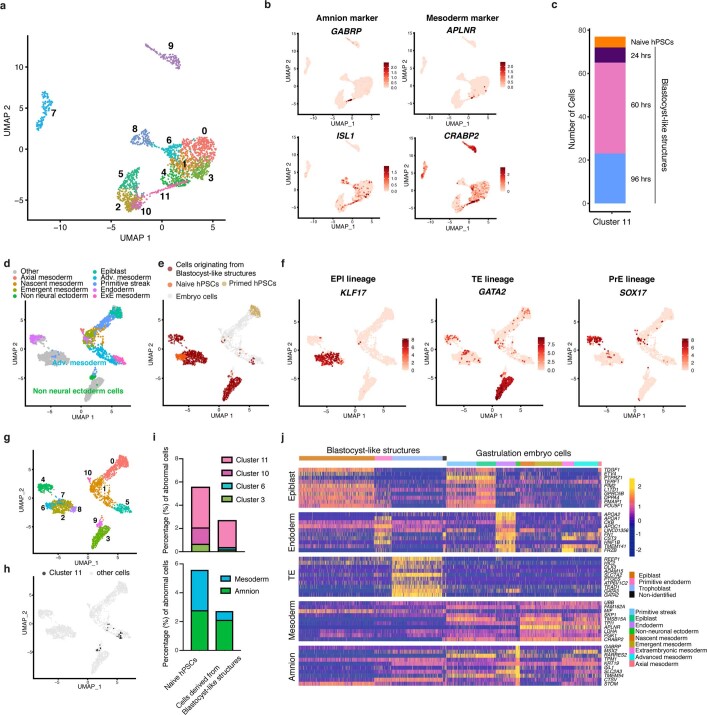
Measurement of generation of off-target cells in human blastocyst-like structures and naive human pluripotent stem cells. **a, b**. UMAP of clusters formed from cells isolated from blastocyst-like structures (high-resolution clustering of 1, x50 as compared to Fig. 2b) (**a**) and displaying the expression levels of genes specific for amnion lineage (**b**). **c**. Origin of the cells composing cluster 11. **d-h**. UMAPs of naive hPSCs, primed hPSCs, cells isolated from blastocyst-like structures and cells isolated from a CS7 staged human embryo. **d**. Coloration of embryo cells based on previously proposed annotations^39^. **e**. Coloration of stem cells based on their origin. **f**. Display of the expression levels of genes specific for each of the three blastocyst lineages (EPI - Epiblast, TE - Trophectoderm, and PrE- Primitive endoderm). **g**. Coloration of individual cells based on their unsupervised cluster affiliation. **h**. Coloration of the cells previously identified as cluster 11 (see **a, b**). **i**. Quantification of the percentage of cells identified as abnormal based on the location in the UMAP in **h** (**top**) and on the cells annotations (**bottom**) for both naive hPSCs (**left**) and cells isolated from blastocyst-like structures (**right**). Similar results were obtained based on the location in the UMAP in (Extended Data Fig. 2c-e). **j**. Heatmap of previously proposed markers of different lineages differentially expressed in cells from blastocyst-like structures and gastrulation-stage embryo^10^. Source data

**Extended Data Fig. 4:**
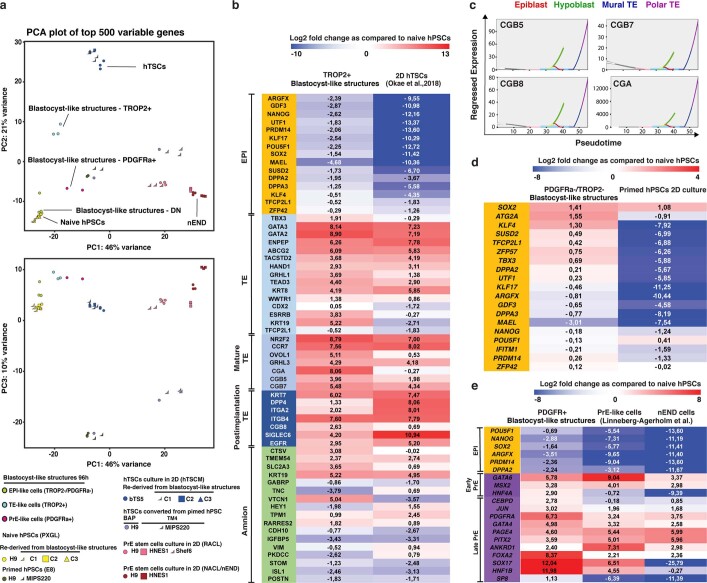
Cells in human blastocyst-like structures are transcriptionally similar to pre-implantation lineages. **a**. Principal component analysis (PCA) plot with PC1 vs PC2 (**top**) or PC1 vs PC3 (**bottom**) computed with top 500 variable gene in the bulk transcriptome of individual lineages of blastocyst-like structures (EPI, TE and PrE); stem cell lines: naive and primed hPSCs; hTSCs: blastocyst derived hTSCs (bTS5)^20^, primed hPSC derived hTSCs (BAP^14^ and TM4 protocols^45^; PrE like stem cell lines (RACL or nEND cells^22^); naive PSC and TSCs rederived from blastocyst-like structures (see methods). **b**. Heatmap of key blastocyst and post-implantation lineage markers differentially expressed between TE analogs (TROP2^+^) of the blastocyst-like structures and hTSCs in their bulk transcriptome. **c**. Pseudotime analysis of human mature TE markers *CGB5, CGB7, CGB8 and CGA*. Gene expression analysis was performed by using the public data analysis tool (https://bird2cluster.univ-nantes.fr/demo/PseudoTimeUI/). **d**. Heatmap of key pluripotency related genes differentially expressed between EPI analogs (PDGFR^-^/ TROP2^-^) in the blastocyst-like structures and primed hPSCs **e**. Heatmap of key pluripotency related genes or PrE markers differentially expressed between PrE analogs (PDGFRα^+^) in the blastocyst-like structures, naive PSC derived PrE-like cells and nEND cells.

**Extended Data Fig. 5:**
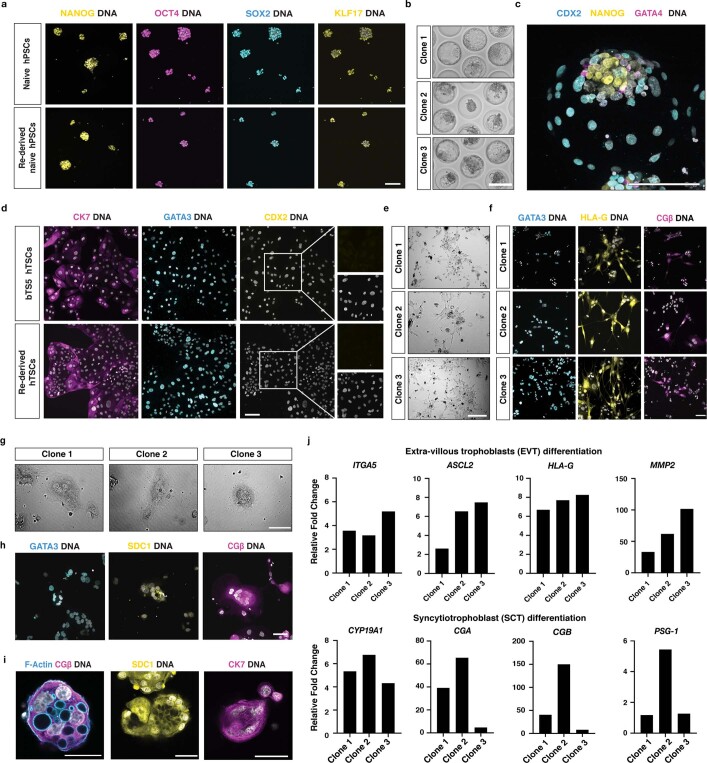
Human blastocyst-like structures are permissive for derivation of stem cell lines. **a**. Immunofluorescence staining for pluripotency factors NANOG (Yellow), OCT4 (Magenta), SOX2 (Cyan) and for naive pluripotency factor KLF17 (Yellow) in naive hPSC controls (**top**) and naive hPSCs derived from blastocyst-like structures (**bottom**). Scale bar: 100 μm. **b**. Phase contrast images of blastocyst-like structures on microwell array formed from three rederived naive hPSC lines. Scale bar: 200 μm. **c**. Immunofluorescence stainings for EPI marker (NANOG), TE marker (CDX2) and primitive endoderm marker (GATA4) in representative second-generation blastocyst-like structures. Scale bar: 100 μm. **d**. Immunofluorescence staining for GATA3 (Cyan), post-implantation trophoblast marker CK7 (Magenta) and CDX2 (Yellow) in bTS5 hTSC (**top**) and hTSCs derived from blastocyst-like structures (**bottom**). Scale bar: 100 μm. **e**. Phase contrast images of day 6 EVT differentiations from three hTSC lines derived from blastocyst-like structures. Scale bar: 150 μm. **f**. Immunofluorescence stainings of trophoblast markers GATA3 (Cyan) and EVT marker HLA-G (Yellow) and CGβ (Magenta) of day 6 EVT analogs from three hTSC lines, derived from blastocyst-like structures. Scale bar: 100 μm. **g**. Phase contrast images of day 3 SCT analogs differentiated from three hTSC lines derived from blastocyst-like structures. Scale bar: 150 μm. **h**. Immunofluorescence stainings for trophoblast markers GATA3 (Cyan) and SCT marker SDC1 (Yellow) and CGβ (Magenta) of day 3 SCT analogs formed from hTSC line derived from blastocyst-like structure (Clone 1). Scale bar: 100 μm. **i**. Immunofluorescence stainings for CGβ (Magenta) counterstained with Phalloidin (Cyan) and Hoechst marking Actin and DNA respectively (**left**), SDC (Yellow), CK7 (Magenta) (**right**) counterstained with Hoechst marking DNA of day 6 trophoblast organoids formed from hTSC lines derived from blastocyst-like structures (Clone 1). Scale bar: 50 μm. **j**. Relative expression levels, as measured by RT-PCR, of day 6 EVT (**top**) and day 3 SCT analogs (**bottom**) with respective undifferentiated hTSCs lines derived from blastocyst-like structures. Expression levels were normalized to expression of *GAPDH*. n=1 biological replicate for three individual clones. Source data

Single-cell transcriptomics analysis showed that blastocyst-like structures formed only three distinct transcriptomic states (Fig. 2a, b, Extended Data Fig. 2a) marked by genes specific to the three founding lineages, including *GATA2* and *GATA3* (TE), *POU5F1* and *KLF17* (EPI), and *GATA4* and *SOX17* (PrE) (Fig. 2c, d, Extended Data Fig. 2b). Comparison with cells from blastocysts, in vitro cultured blastocysts and a gastrulation-stage embryo indicated that the cells in the blastocyst-like structures were transcriptionally similar to the blastocyst stage and distinct from post-implantation stages (Fig. 2e, f, Extended Data Fig. 2c–g). A higher-resolution clustering analysis (from resolution 0.02 to resolution 1) isolated one cluster of non-blastocyst-like cells with a gene-expression pattern reminiscent of post-implantation tissues^15^ (*GABRP*, *ISL1*, *APLNR* and *CRABP2*) (Extended Data Fig. 3a–c) that also appeared transcriptionally similar to amnion (annotated as non-neural ectoderm) and extra-embryonic mesoderm (Extended Data Fig. 3d–j). This sub-population constituted less than 3% of all sequenced cells (Extended Data Fig. 3i). Of note, we found that naive PSC culture also contained 5–6% similarly differentiated cells^19^ (Extended Data Fig. 3i). Bulk RNA-sequencing (RNA-seq) analysis showed that isolated trophoblast analogues (TROP2^+^) had an intermediate transcriptome between those of naive PSCs and post-implantation-like trophoblasts^20^ (TSCs) (Extended Data Fig. 4a). Furthermore, trophoblasts were enriched in blastocyst-stage TE transcripts^11^ (*ESRRB*, *GRHL1*, *OVOL1*, *GATA2*, *GATA3*, *TBX3*, *KRT19*, *CGA*, *CGB5* and *CGB7*) but not in some post-implantation trophoblast markers^11^ (*SIGLEC6* and *DPP4*) (Extended Data Fig. 4b, c). The transcriptome of isolated EPI analogues (TROP2^−^PDGFRa^−^) resembled that of naive PSCs (Extended Data Fig. 4a), was enriched in markers specific for blastocyst-stage EPI^21^ (*KLF17*, *ATG2A*, *SUSD2*, *TFCP2L1*, *DPPA2* and *PRDM14*), and differed from the transcriptome of primed PSCs (Extended Data Fig. 4a, d). Finally, isolated PrE analogues (PDGFRa^+^) had an intermediate transcriptome between those of naive PSCs and extraembryonic endoderm cell lines^22^ (nEND cells) (Extended Data Fig. 4a). PrE analogues were enriched in blastocyst-stage PrE markers (early blastocyst: *GATA6*, *MSX2* and *HNF4A*; late blastocyst: *PDGFRA*, *GATA4*, *SOX17*, *HNF1B* and *FOXA2*) and downregulated in EPI genes (*ARGFX*, *PRDM14*, *SOX2*, *NANOG*, *DPPA2* and *POU5F1*), similar to during blastocyst development^21^ (Extended Data Fig. 4e). Blastocysts have the ability to establish stem cell lines^2^; similarly, blastocyst-like structures enabled de novo derivation of naive PSCs^2^ (NANOG^+^SOX2^+^OCT4^+^KLF17^+^) (Extended Data Fig. 5a) that could form second-generation blastocyst-like structures (Extended Data Fig. 5b, c) and of TSCs^20^ (CDX2^−^GATA3^+^CK7^+^) (Extended Data Fig. 5d) endowed with the capacity for rapid differentiation into syncytio trophoblasts (SCT) and extravillous trophoblasts (EVT) (over 3–6 days; Extended Data Fig. 5e–j). Of note, derivation of PrE cell lines from human blastocysts has not been reported. Thus, blastocyst-like structures formed blastocyst-stage cellular analogues (accounting for more than 97% of the cells sequenced).

## Hippo inhibition is essential

**Fig. 3:**
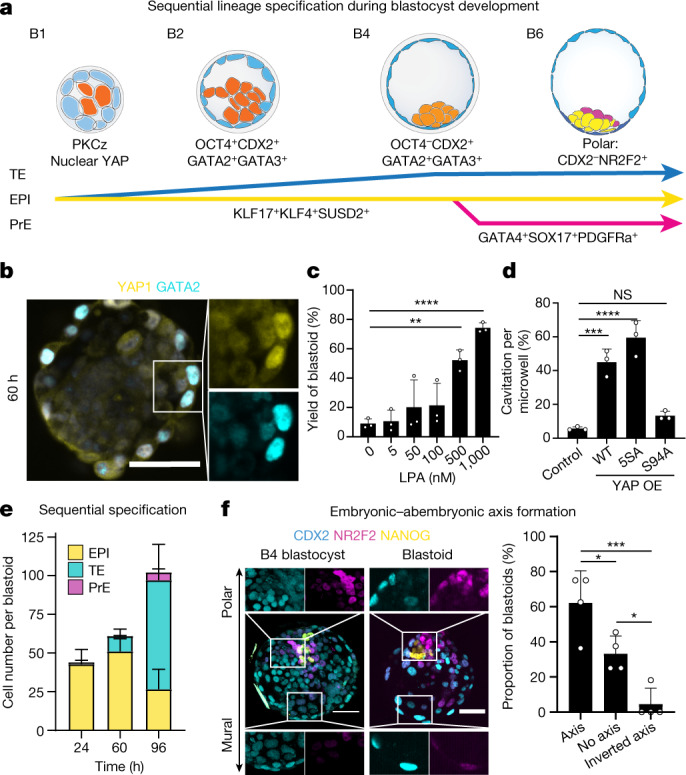
The three lineages form according to the sequence and time of blastocyst development. **a**, Schematic depicting the sequential lineage specification of human blastocysts. **b**, Immunofluorescence of YAP1 (yellow) and GATA2 (cyan) in aggregates of naive PSCs cultured in PALLY medium for 60 h. Scale bar, 50 μm. **c**, Dose-dependent effect of LPA on the yield of blastocyst-like structures. *n* = 3 independent microwell arrays; mean ± s.d.; one-way analysis of variance (ANOVA) and Dunnett’s multiple comparisons test. **P = 0.0016, ****P < 0.0001. **d**, Effect of the overexpression of different variants of YAP1 on cavitation events in early blastocyst-like structures. *n* = 3 experiments; mean ± s.d.; one-way ANOVA and Tukey’s multiple comparisons test. NS, not significant; ***P = 0.0004, ****P = 0.00004. **e**, Total cell numbers per lineage developing blastocyst-like structures at three time points of development (24, 60 and 96 h). Mean ± s.d. EPI: *n* = 11 blastocyst-like structures at 24, 68 and 96 h; TE: *n* = 8 (24 h), *n* = 14 (48 h) and *n* = 15 (96 h) blastocyst-like structures; PrE: *n* = 9 (24 h), *n* = 37 (48 h) and *n* = 9 (96 h) blastocyst-like structures. **f**, Immunofluorescence of CDX2 (cyan), NR2F2 (magenta) and NANOG (yellow) in representative B4-stage human blastocyst (left) and blastocyst-like structures (middle). Quantification of the proportion of blastocyst-like structures with a preferentially polar NR2F2 expression pattern (axis) compared with a preferentially mural NR2F2 expression pattern (inverted axis) (right). *n* = 4 independent experiments with 4–12 blastocyst-like structures in each experiment; mean ± s.d.; one-way ANOVA and Tukey’s multiple comparisons test. *P < 0.05, ***P < 0.001. Scale bar, 50 μm. Source data

**Extended Data Fig. 6:**
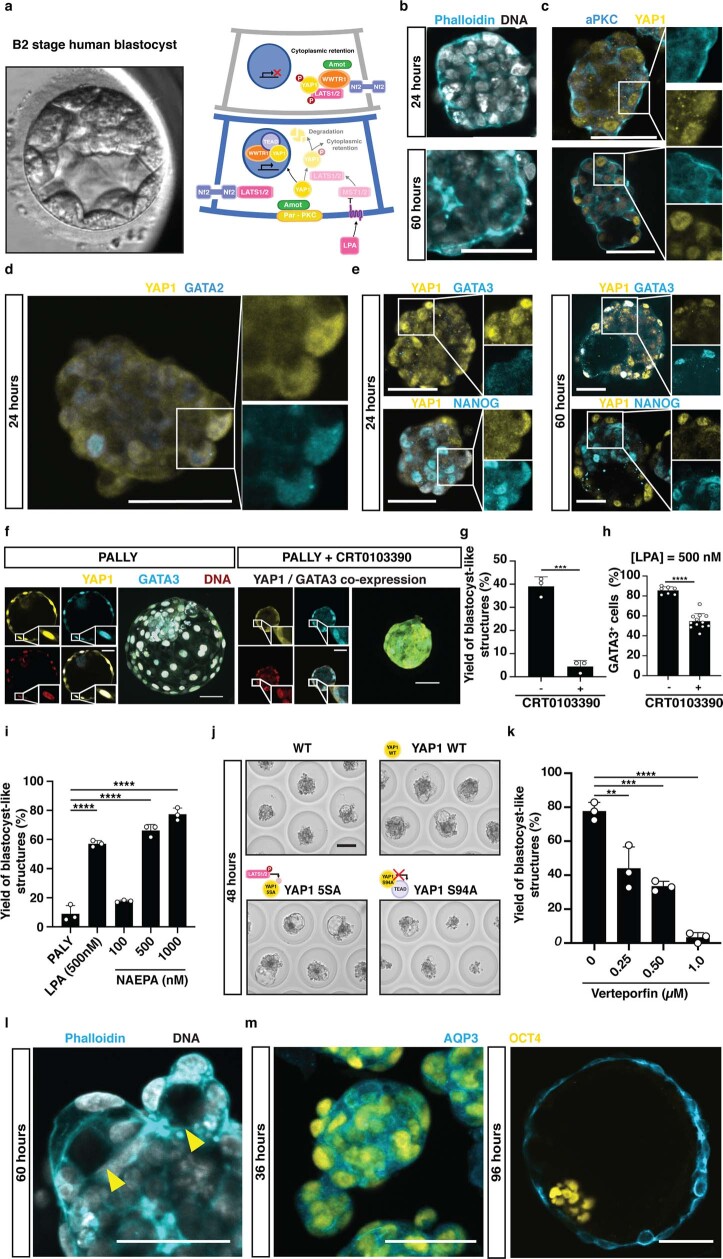
The development of the human trophectoderm analog depends on aPKC and Hippo elements. **a**. A frame from time-lapse microscopy of B2 stage human blastocyst (**left**). Schematic showing the differential Hippo activity in inner and outer cells of developing blastocyst and the molecular regulators of the Hippo signalling pathway (**right**). **b**. Phalloidin fluorescence (Cyan) stainings for F-actin in naive hPSCs aggregates cultured in PALLY medium for 24 h (**top**) and 60 h (**bottom**). Counterstain with Hoechst marking DNA. Scale bar: 50 μm. **c**. Immunofluorescence stainings for aPKC (Cyan) and YAP1 (Yellow) in aggregates of naive hPSCs cultured in PALLY medium for 24 h (**top**) and 60 h (**bottom**). Counterstain with Hoechst marking DNA. Scale bar: 50 μm. **d**. Immunofluorescence stainings for YAP1 (Yellow) with GATA2 (Cyan) in aggregates of naive hPSCs cultured in PALLY medium for 24 h. Scale bar: 50 μm. **e** . Immunofluorescence stainings for YAP1 (Yellow) and GATA3 (Cyan) (**top**) and YAP1 (Yellow) and NANOG (Cyan) (**bottom**) in naive hPSCs aggregates cultured in PALLY medium for 24 h (**left**) and 60 h (**right**). Counterstain with Hoechst marking DNA. Scale bar: 50 μm. **f**. Immunofluorescence staining for YAP1 (Yellow) and GATA3 (Cyan) in blastocyst-like structures cultured without (**top**) or with an aPKC inhibitor (2 μM CRT0103390, **bottom**). Counterstain with Hoechst marking DNA (Red). Insets: Individual and merge channels of YAP1 and GATA3 for a single optical section as well as maximum intensity projection of all the optical sections. Scale bar: 50 μm. **g**. Quantification of the yield of blastocyst-like structures upon the culture in PALLY medium or PALLY medium complemented with an aPKC inhibitor (2 μM CRT0103390). n=3 independent microwell arrays; mean± S.D.; Two tailed unpaired t-test. *** is P=0.0002. **h**. Quantification of the percentage of GATA3^+^ cells in structures cultured in PALLY medium or in PALLY medium complemented with a aPKC inhibitor (2 μM CRT0103390). n=7 blastocyst-like structures for the group cultured in PALLY medium and n=12 aggregates for the group cultured in PALLY medium complemented with CRT0103390. Representative results from three independent experiments. Mean± S.D.; Two-tailed unpaired t-test. **** is P=1.79e-08. **i**. Quantification of the dose dependent effect of the LPA receptor agonist NAEPA on the yield of blastocyst-like structures. The PALY medium (thus without LPA) was complemented with NAEPA. n=3 independent microwell arrays; mean± S.D.; one-way Anova and Tukey’s multiple comparisons test. **** is P<0.0001. **j**. Phase contrast images of representative naive hPSC aggregates cultured in PALLY medium complemented with Doxycycline (100 ng/ml) for 72 h and overexpressing different variants of YAP1. The naive hPSCs aggregates were cultured with an adjusted PALLY medium characterized by a reduced LPA concentration (5 nM). Scale bar: 100 μm. **k**. Measurement of the effect of Verteporfin (suppressor of the YAP1–TEAD complex) on the yield of blastocyst-like structures. n=3 independent microwell arrays; mean± S.D.; one-way Anova and and Dunnett’s multiple comparisons test. ** is p=0.0010, *** is p=0.00019, **** is P<0.0001. **l**. Phalloidin fluorescence staining of F-actin (Cyan) in naive hPSCs aggregates cultured in PALLY medium for 60 h. Counterstain with Hoechst marking DNA. Yellow arrows: Formation of cavities. Scale bar: 50 μm. **m**. Immunofluorescence stainings for Aquaporin3 (AQP3, Cyan) and OCT4 (Yellow) in naive hPSCs aggregates cultured in PALLY medium for 36 (**left**) or 96 h (**right**, blastocyst-like structure stage). Scale bar: 50 μm. Source data

Knowledge about human blastocyst lineage segregation is limited (Fig. 3a). However, inhibition of the Hippo pathway is known to occur in peripheral cells upon acquisition of an apical domain, and is required to initiate TE specification^12^ (Extended Data Fig. 6a). We tested whether blastocyst-like structures co-opted this mechanism. Of note, aPKC and F-actin expression domains appeared co-aligned in outer cells that also accumulated the Hippo downstream effector YAP1 in nuclei (Extended Data Fig. 6b, c). YAP1 nuclear location correlated with GATA2 and GATA3 expression, contrasted with NANOG expression, and became restricted to TE analogues^12^ (Fig. 3b, Extended Data Fig. 6d, e). An aPKC inhibitor (CRT0103390)^12^ largely prevented YAP1 nuclear accumulation, decreased the number of GATA3^+^ cells and prevented the formation of blastocyst-like structures (Extended Data Fig. 6f–h). Conversely, ligands of LPA receptors (LPA and NAEPA) that can inhibit the Hippo pathway enhanced the formation of blastocyst-like structures (Fig. 3c, Extended Data Fig. 6i). Because Hippo pathway inhibition frees YAP1 to enter the nucleus, we tested whether genetically engineered levels and functions of YAP1 could affect morphogenesis. Overexpression of wild-type or constitutively active forms of YAP1 (5SA) accelerated cavitation (Fig. 3d). The interaction between YAP1 and TEAD transcription factors is necessary for downstream gene regulation. Accordingly, over-expression of YAP1 with a mutation in the TEAD binding site (S94A) did not affect cavitation (Fig. 3d, Extended Data Fig. 6j), and verteporfin—which disrupts the YAP1–TEAD interaction—prevented the formation of blastocyst-like structures (Extended Data Fig. 6k). Cavity morphogenesis occurred through the apparent coalescence of multiple fluid-filled cavities^23^ (Extended Data Fig. 6l). Aquaporin 3 (AQP3), the water transporter most highly expressed in human blastocysts^11^, was initially visible in all cells (36 h) and was then restricted to TE analogues (96 h) (Extended Data Fig. 6m). Thus, similar to human blastocysts^12^, TE specification and morphogenesis within these structures depends on aPKC, inhibition of the Hippo pathway, nuclear translocation of YAP1 and the ability of YAP1 to bind TEAD transcription factors.

## Adequate developmental sequence

**Extended Data Fig. 7:**
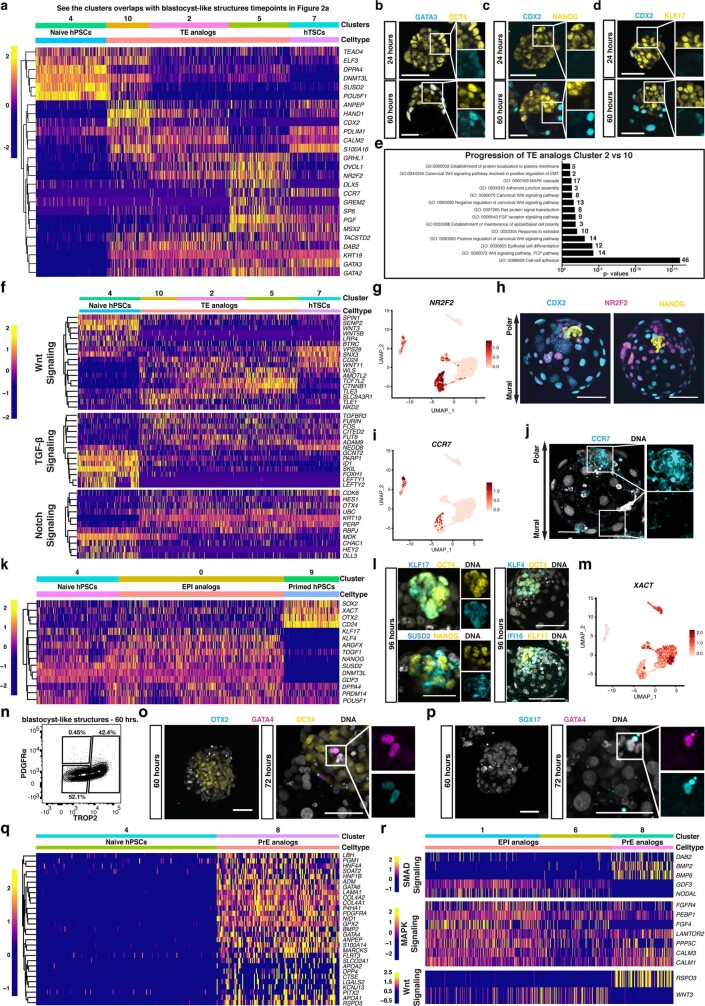
Blastocyst-like structures recapitulate the sequential specification of lineages occurring during blastocyst development. **a**. Heatmap of the average count values in the expression of TE genes upon formation of the blastocyst-like structures TE analogs. **b-d**. Immunofluorescence stainings for GATA3 (Cyan)) and OCT4 (Yellow) **(b)** or CDX2 (Cyan) and NANOG (Yellow) **(c)** or CDX2 (Cyan) and KLF17 (Yellow) **(d)** in naive hPSCs aggregates cultured in PALLY medium for 24 h **(top)** or 60 h **(bottom)**. Scale bar: 50 μm. **e**. Gene ontology terms associated with the genes differentially regulated in the late TE analog of blastocyst-like structures (cluster 10) as compared to the early TE (cluster 2). **f**. Heatmap of average count values of Wnt, TGF-β and Notch signaling-associated genes in cells from cluster 4 (naive hPSCs), 10, 2 and 5 (TE analogs) and 7 (TSC). **g**. UMAPs of single cells isolated from blastocyst-like structures and displaying the expression levels of polar trophectoderm specific gene: NR2F2. **h**. Immunofluorescence staining for CDX2 (Cyan), NR2F2 (Magenta) and NANOG (Yellow) in blastocyst-like structures. Scale bar: 100 μm. **i**. UMAPs of single cells isolated from blastocyst-like structures and displaying the expression levels of polar trophectoderm specific gene: CCR7. **j**. Immunofluorescence stainings for CCR7 (Cyan) in a blastocyst-like structures. Counterstain with Hoechst marking DNA. Scale bar: 50 μm. **k**. Heatmap of average count values of top differentially regulated genes in cells from cluster 4 (naive hPSCs), 0 (EPI analogs) and 9 (primed hPSCs). **l**. Immunofluorescence staining for KLF17 (Cyan) and OCT4 (Yellow) or KLF4 (Cyan) and OCT4 (Yellow) **(top)** and SUSD2 (Cyan) and NANOG (Yellow) or IFI16 (Cyan) and KLF17 (Yellow) **(bottom)** in blastocyst-like structures. Counterstain with Hoechst marking DNA. Scale bar: 100 μm. **m**. UMAPs of single cells isolated from blastocyst-like structures and displaying the expression levels of X chromosome activation-related gene-*XACT*. **n**. Flow cytometry analysis plot of cells isolated from blastocyst-like structures cultured in PALLY medium for 60 h and stained for lineage-specific surface markers PDGFRa (PrE) and TROP2 (TE). **o, p**. Immunofluorescence stainings for OTX2 (Cyan), GATA4 (Magenta) and OCT4 (Yellow) (**o)** and SOX17 (Cyan) and GATA4 (Magenta) (**p**) in naive hPSCs aggregates cultured in PALLY medium for 60 h. Counterstain with Hoechst marking DNA. Scale bar: 50 μm. **q**. Heatmap of the average count values in the expression of PrE genes upon formation of the blastocyst-like structures PrE analogs. **r**. Heatmap of average count values of SMAD, MAPK and Wnt signaling-associated genes in cells from cluster 1, 6 (EPI analogs) and 8 (PrE analogs). Source data

In blastocysts, TE (GATA2^+^DAB2^+^) and EPI (KLF17^+^NANOG^+^) cells appear first^11,21^ (5–6 dpf) and PrE cells (GATA6^+^ADM^+^) and polar TE cells (pTE) (CDX2^−^NR2F2^+^) appear last^21^ (6–7 dpf). This sequence is recapitulated in the blastocyst-like structures. Trophoblasts (*DAB2*^+^, *CDX2*^+^, *GATA2*^+^, *GATA3*^+^) formed first (within 24 h) (Fig. 3e, Extended Data Fig. 7a), and changed the levels of transcripts related to PKC and Hippo signalling (*AKAP12*, *CAPZB*, *ULK4*, *MOB1A*, *AMOT*, *AMOTL2*, *LATS2* and *TEAD1*) (Supplementary Table 1). At protein level, early TE-like cells were first YAP1^nuclear^GATA2^+^ (at 24 h) and then CDX2^+^GATA3^+^, while maintaining expression of KLF17 and OCT4, but not NANOG (at 60 h) (Extended Data Fig. 7b–d). Subsequently, OCT4 became undetectable^11^ (Fig. 1g, Extended Data Fig. 1i). Genes associated with SMAD, ERK, Notch and Wnt signaling pathways were regulated during this process (Extended Data Fig. 7e, f, Supplementary Table 1). Finally, pTE analogues matured as marked by expression of *OVOL1*, *GREM2*, *CCR7*, *SP6* and *NR2F2* (Extended Data Fig. 7g–j), upregulation of NR2F2 and CCR7^11^ and downregulation of CDX2 (Fig. 3f, Extended Data Fig. 7h, j). The transcriptome of EPI analogues maintained core blastocyst markers (*POU5F1*, *NANOG*, *KLF17*, *SUSD2*, *KLF4*, *ARGFX* and *GDF3*) (Fig. 3e, Extended Data Fig. 7k, l, Supplementary Table 1), while undergoing a progression characterized by regulation of Nodal (*NODAL*, *LEFTY1* and *LEFTY2*) and mTOR (*LAMTOR1*, *LAMTOR4*, *LAMTOR5*, *XBP1P1* (*XBP1*, also known as) *SEC13* and *MLST8*) signalling-related genes, and of the X chromosome activation-related gene *XACT* (Extended Data Fig. 7k–m, Supplementary Table 1, cluster 4 versus cluster 0). At the protein level, EPI analogues were marked by KLF17 and SUSD2, which are specifically highly expressed at the blastocyst stage (Extended Data Fig. 7l). PrE analogues appeared within 60 h and GATA4, OTX2 and SOX17 were detected^11^ within 72 h (Fig. 3e, Extended Data Fig. 7n–p). Early PrE marker genes^21^ (*GATA6*, *LBH*, *ADM* and *LAMA1*) were uniformly expressed among the PrE analogues, while some late PrE marker genes (*CTSE*, *APOA1*, *PITX2* and *SLCO2A1*) were expressed only in a subpopulation of cells, suggesting a progression toward the late blastocyst stage^11^ (Extended Data Fig. 7q). By 96 h, mature PrE analogues had regulated SMAD (*NODAL*, *BMP2*, *BMP6*, *GDF3*, *ID1* and *IDI2*) and Wnt signalling-related transcripts (*WNT3*, *RSPO3* and *LBH*) and were enriched in transcripts controlling extracellular matrix organization (*LAMA1*, *LAMB1*, *LAMC1*, *COL4A1* and *COL4A2*), and endodermal and epithelial differentiation (Extended Data Fig. 7q, r, Supplementary Table 1, cluster 6 versus cluster 8). Because this model morphologically resembles the human blastocyst (see criteria in Methods), efficiently generates analogues of the three lineages with transcriptomes matching the blastocyst stage, and forms these analogues according to the sequence and approximate pace of blastocyst development, we refer to it as a human blastoid.

## Distinct attachment to endometrial cells

**Fig. 4:**
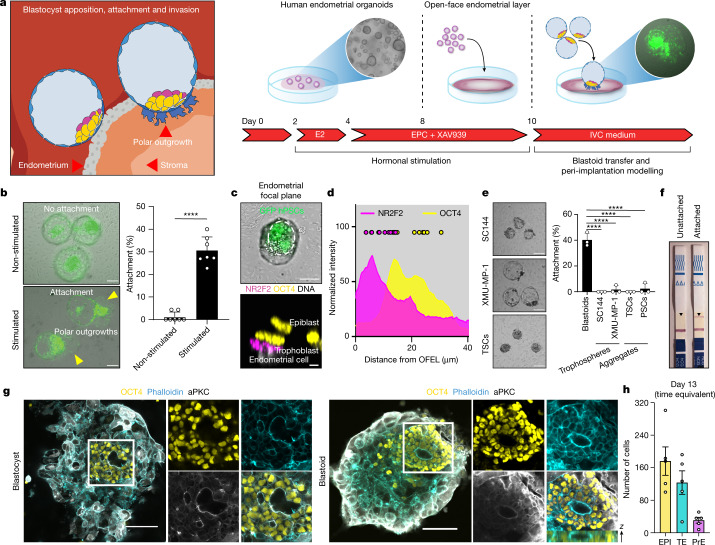
Human blastoids recapitulate aspects of implantation. **a**, Left, schematic of the modelled implantation process. Right, OFEL priming assay using EPC/XAV939. E2, β-oestradiol; EPC, E2 + progesterone + cAMP. **b**, Representative phase-contrast images of blastoids (GFP^+^) 24 h after deposition onto non-stimulated (top left) or stimulated (bottom left) OFELs. Scale bar, 100 μm. Attachment efficiency of human blastoids (right). *n* = 7 independent experiments from 3 different donors; mean ± s.d.; unpaired two-tailed *t*-test. ****P = 4.5 × 10^−8^. **c**, Representative images of recently attached human blastoids (12 ± 4 h). Top, the dashed delineates the inner cluster of blastoids formed from GFP^+^ naive PSCs (also see Supplementary Video 3). Scale bar, 100 μm. Bottom, *x*–*z* plane of NR2F2 (magenta) and OCT4 (yellow) immunofluorescence in blastoids immediately after attachment. Scale bar, 5 μm. **d**, Intensity profile of immunofluorescence of NR2F2 and OCT4 in blastoids immediately after attachment. *n* = 10. **e**, Left, representative phase-contrast images of trophospheres formed using 3 μM SC144 (top) or 2 μM XMU-MP-1 (middle), and aggregates of TSCs (bottom) deposited onto stimulated OFELs. Scale bar, 100 μm. Right, attachment efficiency. *n* = 3 independent experiments; mean ± s.d.; one-way ANOVA and Dunnett’s multiple comparisons test. ****P < 0.0001. **f**, Pregnancy test strips detecting secretion of CGβ into the medium of unstimulated OFELs with unattached blastoids and stimulated OFELs with attached blastoids (48 h on OFEL; see ELISA assay in Extended Data Fig. 10b). **g**, Immunofluorescence of OCT4 (yellow) and aPKC (grey) in human blastocysts (left) or blastoids (right) grown in post-implantation culture condition for 4 days, counterstained with phalloidin marking F-actin (cyan). Scale bars, 100 μm. **h**, Number of cells positive for OCT4, GATA3 and GATA4 in blastoids grown in post-implantation culture for 6 days (time equivalent, day 13). *n* = 5. mean ± s.d. Source data

**Extended Data Fig. 8:**
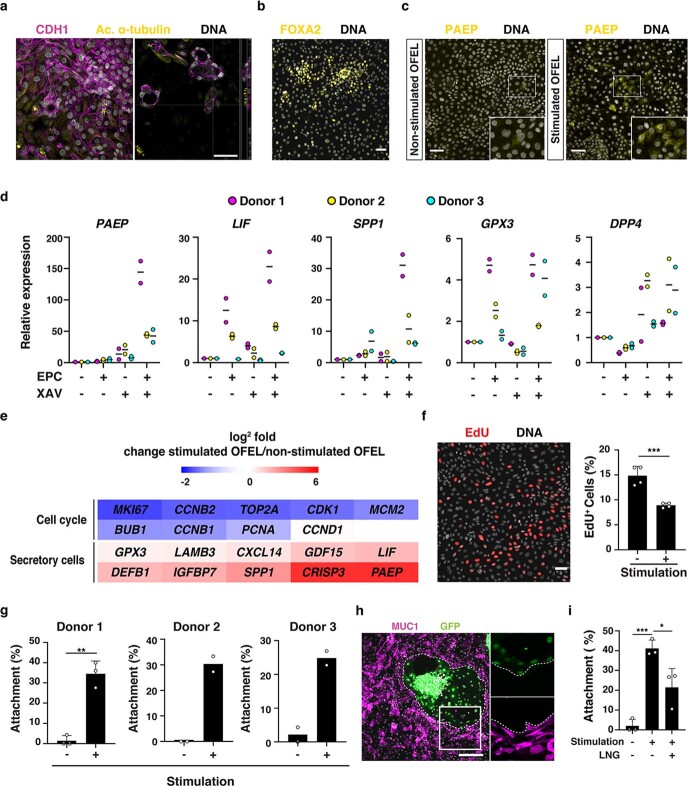
Human blastoids recapitulate aspects of implantation. **a**. Immunofluorescence stainings for CDH1 (Magenta) and a ciliated cell marker acetylated α-tubulin (Yellow) in OFELs (**left**). Y-Z plane shows the apical location of the cilia (**right**). Scale bar: 50 μm. **b**. Immunofluorescence staining for FOXA2 (Yellow) marking the endometrial glandular cells in OFELs. Scale bar: 50 μm. **c**. Immunofluorescence staining for PAEP (Yellow) in non-stimulated (**left**) and stimulated (**right**) OFELs. **d**. qRT-PCR measurement of the expression levels of window-of-implantation markers in OFELs cultured with different media. Ctrl: Control medium, E: Estradiol, P: Progesterone, C: cAMP, X: XAV-939. Expression levels were normalized relative to the housekeeping gene GAPDH and the control condition. n = 2 independent experiments. The colors depict the data from 3 different donors. **e**. Heatmap of key cell cycle and secretory epithelial genes differentially expressed between stimulated and non-stimulated OFELs in bulk transcriptome. **f**. Staining for incorporated EdU (Red) reflective of cell proliferation in a stimulated OFEL (**left**). Scale bar: 50 μm. Quantification of the number of EdU^+^ cells in non-stimulated and stimulated OFELs (**right**). Counterstain with Hoechst marking DNA. n=4 independent experiments. mean± S.D.; Unpaired two-tailed *t*-test, *** is P = 0.0009. **g**. Quantification of blastoid attachment onto OFELs prepared using endometrial organoids from 3 different donors. n=3 independent experiments for donor 1 and n=2 independent experiments for donor 2 and 3; mean± S.D.; Unpaired two-tailed *t*-test, ** is P =0.0011. **h**. Immunofluorescence stainings for MUC1 (Magenta), a glycoprotein that highly expresses at the luminal epithelial surface of endometrium in the receptive phase^46^, with an attached GFP+ blastoid (48 h after deposition onto an OFEL). Dashed lines indicate the area that trophoblast cells repelled endometrial cells. Scale bar: 200 μm **i**. Quantification of blastoid attachment onto non-stimulated, stimulated OFELs, and OFELs additionally exposed to the contraceptive Levonorgestrel (LNG, 10 μM). n=3 independent experiments. mean± S.D.; one-way Anova and Tukey’s multiple comparisons test, * is P = 0.0211, *** is P = 0.0006. Source data

At 7 dpf, the human blastocyst initiates implantation in utero through the attachment of its TE to a receptive endometrium (Fig. 4a, left). We tested whether blastoids could model this interaction by seeding endometrial organoids^24^ in 2D to form an open-faced endometrial layer (OFEL) to facilitate the deposition of blastoids (Fig. 4a, right). Subpopulations of the OFEL were positive for acetylated α-tubulin, marking ciliated epithelial cells^24^ (Extended Data Fig. 8a), and FOXA2, marking glandular epithelial cells (Extended Data Fig. 8b). The window of implantation is the period during which the uterus becomes receptive for blastocyst implantation. It opens upon exposure to oestrogen (E2) and progesterone (P4), and correlates with regulation of Wnt signalling^25^. Accordingly, OFELs responded to E2, P4, cAMP and XAV939 by upregulating the expression of genes that mark the mid-secretory-phase endometrium (Extended Data Fig. 8c–e) and decreasing proliferation, which are hallmarks of receptivity^25^ (Extended Data Fig. 8e, f). Notably, blastoids deposited onto non-stimulated OFELs did not attach; however, they did attach to and repel the endometrial cells of stimulated OFELs, as occurrs in utero (Fig. 4b, Extended Data Fig. 8g, h). The contraceptive levonorgestrel impaired blastoid attachment (Extended Data Fig. 8i). We concluded that human blastoids are capable of interacting specifically with endometrial cells that have been made receptive.

## Epiblast signals gatekeep trophectoderm attachment

**Extended Data Fig. 9:**
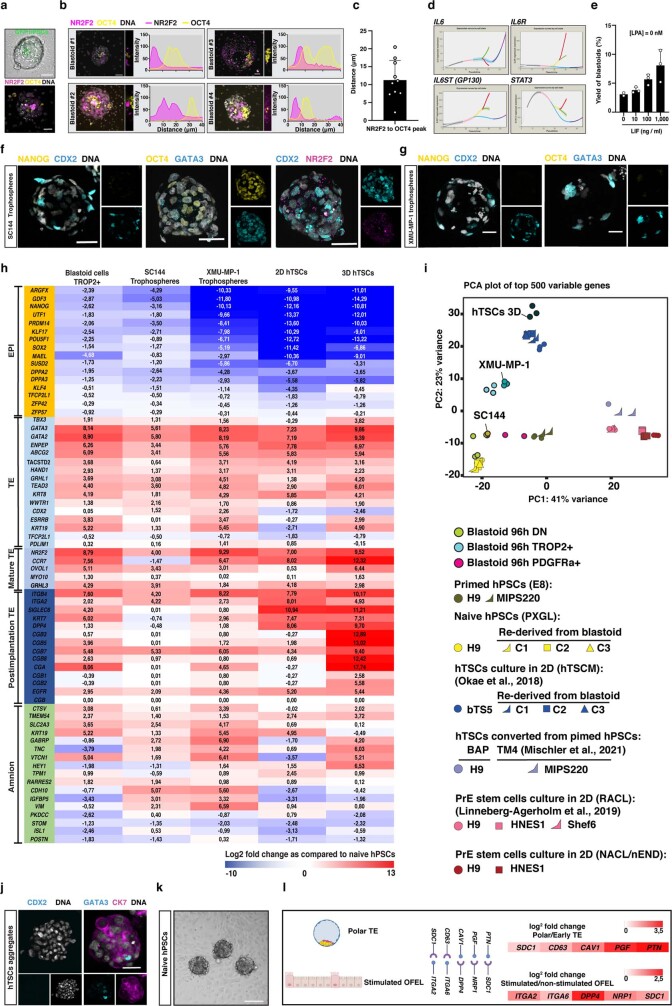
Trophectoderm state is crucial for interaction with endometrium during implantation. **a**. Representative images of human blastoids shortly after attachment to an OFEL. Dotted line outlines the inner cluster of blastoids that were formed using GFP^+^ naive hPSCs (**top**, also see Supplementary Video 3). Immunofluorescence stainings for NR2F2 (Magenta) and OCT4 (Yellow) in blastoids shortly after attachment to an OFEL (**bottom**). **b**. Immunofluorescence stainings for NR2F2 (Magenta) and OCT4 (Yellow) and respective fluorescence intensity profiles of representative blastoids immediately after attachment onto OFEL. Profiles were measured perpendicular to the plane of attachment (**right**). Line width, 10 µm. Y axis shows normalized intensity. **c**. Quantification of the distance between the first peak of fluorescence intensity profiles of NR2F2 and OCT4. n=10 attached blastoids. mean± S.D. **d**. Pseudotime analysis of human pre-implantation development showing the expression of *IL6*, *IL6R*, *GP130* and *STAT3*. Gene expression analysis is performed by using the public data analysis tool (https://bird2cluster.univ-nantes.fr/demo/PseudoTimeUI/). **e**. Quantification of the dose dependent effect of LIF on the yield of blastoids. n=2 (without Lif) and n=3 (all other conditions) independent experiments. mean± S.D. **f**. Immunofluorescence staining for NANOG (Yellow) and CDX2 (Cyan) (**left**), OCT4 (Yellow) and GATA3 (Cyan) (**middle**) and CDX2 (Cyan) and NR2F2 (Magenta) (**right**) in representative trophospheres formed from a blastoid exposed to SC144. Scale bar: 50 μm. **g**. Immunofluorescence staining for NANOG (Yellow) and CDX2 (Cyan) (**left**), OCT4 (Yellow) and GATA3 (Cyan) (**right**) in representative trophospheres formed from a blastoid exposed to XMU-MP-1. Scale bar: 50 μm. **h**. Heatmap of key lineage specific genes differentially expressed in bulk transcriptome of the trophectoderm of blastoids (TROP2 positive cells), trophospheres (SC144 or XMU) and TSCs (2D or 3D) compared to naive hPSCs. **i**. PCA plot computed using bulk transcriptome of blastoid cells, hPSCs (naive, primed or blastoid rederived naive cell lines), TSCs (bTS5, blastocyst rederived lines or human stem cell derived TSC like cells) and pluripotent stem cell derived primitive endoderm like cells (RACL or NACL cells). **j**. Immunofluorescence stainings for CDX2 (Cyan) (**left**) and CK7 (Magenta) and GATA3 (Cyan) (**right**) in aggregates formed from bTS5 hTSCs. Counterstain with Hoechst marking DNA. Scale bar: 50 μm. **k**. Representative phase contrast images of aggregates of naive hPSCs, deposited onto stimulated OFELs. Scale bar: 100 μm. **l**. List of selected putative ligand-receptor pairs involved in cross-talk between polar trophectoderm and endometrial epithelial cells. The list was generated by in silico ligand receptor analysis of genes enriched in polar trophectoderm and stimulated OFEL, using Cellinker^47^. Source data

Human blastocysts attach to the endometrium via the pTE, which is defined by its contact with the EPI. Similarly, blastoids reproducibly initiated attachment through this region (Fig 4c, d, Extended Data Fig. 9a–c, Supplementary Videos 3, 4). We tested the role of the pTE–EPI interface by forming trophospheres devoid of EPI. IL-6 is highly expressed in the pTE and transcripts for its receptor (*IL6R* and *IL6ST* (also known as *GP130*)) and effector (*STAT3*) are present at high levels in the EPI (Extended Data Fig. 9d). Consistent with a role for STAT signalling in the EPI, the efficiency of blastoid formation increased with LIF concentration (Extended Data Fig. 9e), whereas the addition of a GP130 inhibitor (SC144) yielded trophospheres (Fig. 4e, Extended Data Fig. 9f). The presence of a potent inhibitor of the Hippo kinases MST1 and MST2 (XMU-MP-1) also yielded trophospheres (Fig. 4e, Extended Data Fig. 9g). The transcriptomes of these trophospheres reflected early and late blastocyst-stage TE (Extended Data Fig. 9h, i). Neither type of trophosphere attached to OFELs (Fig. 4e), and nor did aggregates of TSCs^20^ that reflect post-implantation cytotrophoblasts^26^ (CDX2^−^CK7^+^) or aggregates of naive PSCs (Fig. 4e, Extended Data Fig. 9j, k). We thus conclude that signals from the EPI induce pTE maturation and endows it with the potential to interact with endometrial cells. This potential appears lost in TSCs reflecting a post-implantation stage. On the basis of transcriptome analysis and in utero data^25^, we propose several pairs of molecules whose transcripts became more abundant upon endometrial cell stimulation and pTE analogue maturation (Extended Data Fig. 9l)s that might mediate the first contact between blastocyst and uterus. Overall, we conclude that a polar-like TE state, whose maturation depends on EPI inductions, gatekeeps the interaction of the blastocyst with the endometrium. This interaction and subsequent maturation create a window of opportunity for blastocyst implantation.

## Modelling post-stages on day 13

**Extended Data Fig. 10:**
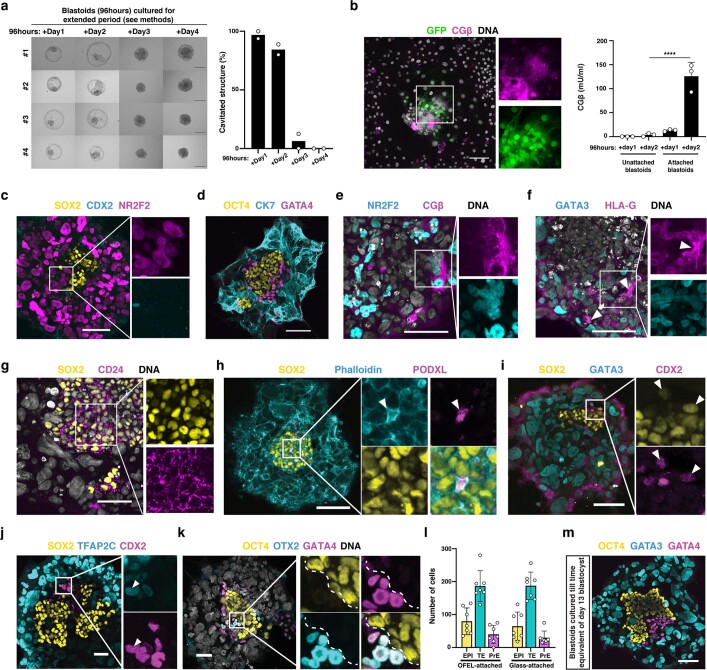
Human blastoids recapitulate aspects of peri-implantation progression until day 13. **a**. Bright-field images of human blastoids (96 h) cultured for 4 additional days on a low attachment plate in post implantation culture condition (**left**). Each row shows a time series of an individual blastoid for 4 days. Note that, blastoids stably retain cavities at least for 2 days upon transferring to IVC media which has different osmolarity compared to the N2B27 media with PALLY. (See the methods for the composition of post implantation culture media.) Scale bar: 200 μm. Quantification of percentage of blastoids retaining cavities on each day of postimplantation stage culture (**right**). n=2 independent experiments. **b**. Immunofluorescence staining for the syncytiotrophoblast-associated marker CGβ (Magenta) in GFP^+^ blastoids attached onto stimulated OFELs (48 h after deposition) (**left**). Counterstain with Hoechst marking DNA. Scale bar: 50 μm. ELISA measurements of the concentration of the protein CGβ secreted into the culture medium of unstimulated OFELs with unattached blastoids and stimulated OFELs with attached blastoids (24 and 48 h) (**right**). n=3 independent experiments. mean± S.D.; one-way Anova and Tukey’s multiple comparisons test, **** is P = 0.00006. **c**. Immunofluorescence stainings for CDX2 (Cyan), NR2F2 (Magenta) and SOX2 (Yellow) in blastoids grown in postimplantation culture condition for 4 days. Scale bar: 100 μm. **d**. Immunofluorescence stainings for OCT4 (Yellow), CK7 (Cyan) and GATA4 (Magenta) in blastoids grown in postimplantation culture condition for 4 days. Scale bar: 100 μm. **e, f**. Immunofluorescence stainings for CGβ (Magenta) and NR2F2 (Cyan) (**e**) or HLA-G (Magenta) and GATA3 (Cyan) (**f**), in blastoids grown in postimplantation culture condition for 4 days (**e**) or 6 days (**f**). Counterstain with Hoechst marking DNA. Arrowhead points HLA-G positive EVT like cells. Scale bar: 100 μm. **g**. Immunofluorescence stainings for CD24 (Magenta) and SOX2 (Yellow) in blastoids grown in postimplantation culture condition for 6 days. Counterstain with Hoechst marking DNA. Scale bar: 100 μm. **h**. Immunofluorescence stainings for PODXL (Magenta) and SOX2 (Yellow) in blastoids grown in postimplantation culture condition for 4 days. Counterstain with Phalloidin marking F-actin (Cyan). Arrowhead points pro-amniotic-like cavity. Scale bar: 100 μm. **i-k**. Immunofluorescence stainings for SOX2 (Yellow), GATA3 (Cyan) and CDX2 (Magenta) (**i**), SOX2 (Yellow), CDX2 (Magenta) and TFAP2C (Cyan) (**j**), OCT4 (Yellow), GATA4 (Magenta) and OTX2 (Cyan) (**k**) in blastoids grown in postimplantation culture condition for 4 days. Counterstain with Hoechst marking DNA. Scale bar: 100 μm. **l**. Quantification of number of cells belonging to EPI, TE or PrE lineages in the blastoids cultured in postimplantation culture condition for four days on glass or OFEL. n=7 biological replicates. mean± S.D. **m**. Immunofluorescence stainings for OCT4 (Yellow), GATA3 (Cyan) and GATA4 (Magenta) in blastoids grown in postimplantation culture condition for 6 days corresponding to time equivalent of day 13 of cultured human blastocyst (**left**). Scale bar: 100 μm. Source data

The blastoid morphology was stable for two days in peri-implantation culture conditions^27,28^ (Extended Data Fig. 10a). Clinical pregnancy is characterized by the detection of chorionic gonadotropin-β hormone (CGβ). Accordingly, upon attachment, blastoids formed trophoblasts expressing CGβ at levels detectable using standard pregnancy tests and ELISA (Fig. 4f, Extended Data Fig. 10b). NR2F2^+^ pTE analogues proliferated and decreased CDX2 expression while upregulating the peri-implantation gene cytokeratin 7 (*KRT7* (a.k.a.*CK7*)) (Extended Data Fig. 10c, d). Some trophoblasts further differentiated into SCT and EVT expressing CGβ and HLA-G, respectively (Extended Data Fig. 10e, f). EPI analogues maintained expression of OCT4 and SOX2, upregulated the primed pluripotency marker CD24 (Fig. 4g, Extended Data Fig. 10g) and patterned cortical F-actin as during post-implantation EPI epithelization, and some blastoids cultured in vitro for 4 days past the equivalent of the blastocyst stage (day 7) formed pro-amniotic-like cavities enriched with F-actin, PODXL and aPKC (Fig. 4g, Extended Data Fig. 10h). A subpopulation in the periphery of the EPI analogue expressed CDX2 along with SOX2 or TFAP2C, suggestive of early amnion analogues (Extended Data Fig. 10i, j). Extra-embryonic endoderm analogues were characterized by restricted expression of OTX2^11^ (Extended Data Figs. 7o, 10k). Upon prolonged culture (up to 6 days), the three lineages consistently expanded (Fig. 4h, Extended Data Fig. 10l, m) until a time equivalent of day 13, although, similar to blastocysts, their organization did not reflect that developmental stage.

## Discussion

Human blastoids morphologically resemble the human blastocyst (criteria described in Methods), efficiently generate analogues of its three lineages with transcriptomes matching the human blastocyst stage, and form these analogues according to the sequence (TE and EPI, then pTE and PrE) and approximate pace (4 days) of blastocyst development. We therefore propose that this model is relevant for the study of human blastocyst development and implantation. Some initial parameters and end-point criteria that are useful to form and define these models^5–9^ are summarized in Supplementary Table 2. Mimicking the interaction between the epiblast and trophectoderm revealed that the epiblast induces the local maturation of polar trophectoderm and subsequently endows it with the capacity to attach onto stimulated endometrial cells. In future, human blastoids may be used to help identify therapeutic targets and contribute to preclinical modelling (for example, in vitro fertilization medium complements such as LPA and NAEPA or contraceptives such as SC144 (ref.^3^)). Considering the proportionality (balancing the benefits and harms) and subsidiarity (pursuing goals using the morally least problematic means) of human embryology, blastoids represent an ethical opportunity to complement research using embryos^4^.

## Methods

### Ethical approvals

The use of human embryos donated to research as surplus of IVF treatment was allowed by the French embryo research oversight committee (Agence de la Biomédecine) under approval number RE13-010 and RE18-010. All human pre-implantation embryos used in this study were obtained following informed consent from the couples who donated embryos and cultured at the Assisted Reproductive Technology unit of the University Hospital of Nantes, France, which are authorized to collect embryos for research under approval number AG110126AMP of the Agence de la Biomédecine. Human endometrium samples were obtained from patients who signed an informed consent form and protocols approved by the Ethics Committee of Royan Institute (IR.ACECR.ROYAN.REC. 1397.93) and of Shahid Beheshti University of Medical Sciences (IR.SBMU.MSP.REC. 1396.25). The Wicell line H9 was used under the agreement 20-WO-341 for a research program entitled ‘Modeling early human development: Establishing a stem cell based 3D in vitro model of human blastocyst (blastoids)’. Blastoid generation was approved by the Commission for Science Ethics of the Austrian Academy of Sciences. This work did not exceed a developmental stage normally associated with 14 consecutive days in culture after fertilization even though this is not forbidden by the ISSCR Guidelines as far as embryo models are concerned. All experiments complied with all relevant guidelines and regulations, including the 2021 ISSCR guidelines that forbid the transfer of human blastoids into any uterus^4^.

### Culture of human naive pluripotent stem cells

Experiments were done using the following PSC lines; human ES cell lines: H9, Shef6 and HNES1. Induced pluripotent stem cell (iPSC) lines: cR-NCRM2 and niPSC 16.2.b. The H9 and H9-GFP lines reset to the naive state were provided by the laboratory of Y. Takashima. Other naive human ES cells and iPSCs were provided by the laboratory of A. Smith. Naive PSCs were cultured on gelatin-coated plates including a feeder layer of gamma-irradiated mouse embryonic fibroblasts (MEFs) in PXGL medium, as previously reported^29^. PXGL medium is prepared using N2B27 basal medium supplemented with PD0325901(1 µM, MedChemExpress, HY-10254), XAV-939 (1 µM, MedChemExpress, HY-15147), Gö 6983 (2 µM, MedChemExpress, HY-13689) and human leukemia inhibitory factor (hLIF, 10 ng ml^−1^, in-house made) as previously reported^29^. N2B27 basal medium contained DMEM/F12 (50%, in house made), neurobasal medium (50%, in-house made), N-2 supplement (Thermo Fisher Science, 17502048), B-27 supplement (Thermo Fisher Science, 17504044), GultaMAX supplement (Thermo Fisher Science, 35050-038), non-essential amino acid, 2-mercaptoethanol (100 µM, Thermo Fisher Science, 31350010), and bovine serum albumin solution (0.45%, Sigma-Aldrich, A7979-50ML). Cells were routinely cultured in hypoxic chambers (5% CO_2_, 5% O_2_) and passaged as single cells every three to four days. All cell lines had routinely tested negative for mycoplasma.

### Culture of primed pluripotent embryonic stem cells

Primed H9 cells were cultured on Vitronectin XF (STEMCELL Technologies, 07180) coated plates (1.0 μg cm^−2^) using Essential 8 medium (prepared in-house).

### Microwell arrays

Microwell arrays comprising microwells of 200 µm diameter were imprinted into 96-well plates as previously described^30,31^.

### Induction of blastoids and trophospheres

Naive PSCs were treated with Accutase (Biozym, B423201) at 37 °C for 5 min, followed by gentle mechanical dissociation with a pipette. After centrifugation, the cell pellet was resuspended in PXGL medium, supplemented with Y-27632 (10 µM, MedChemExpress, HY-10583). To exclude MEFs, the cell suspension was transferred onto gelatin-coated plates and incubated at 37 °C for 70 min. After MEF exclusion, the cell number was determined using a Countess automated cell counter (Thermo Fisher Scientific) and trypan blue staining to assess cell viability. The cells were then resuspended in N2B27 medium containing 10 µM Y-27632 (aggregation medium) and 3.0 × 10^4^ cells were seeded onto a microwell array included into a well of a 96-well plate and placed in a hypoxic chamber (5% CO_2_, 5% O_2_) for the whole period of blastoid or trophosphere formation. The cells were allowed to form aggregates inside the microwell for a period ranging from 0 to 24 h depending on the cell lines and based on their propensity for aggregation. Subsequently, the aggregation medium was replaced with PALLY medium (N2B27 supplemented with PD0325901 (1 µM), A 83-01 (1 µM, MedChemExpress, HY-10432), 1-oleoyl lysophosphatidic acid sodium salt (LPA)^32^ (500 nM, Tocris, 3854), hLIF (10 ng ml^−1^) and Y-27632 (10 µM)). The PALLY medium was refreshed every 24 h. After 48 h, the PALLY medium was replaced with N2B27 medium containing 500 nM LPA and 10 µM Y-27632. At 96 h, a blastoid is defined based (1) on morphological similarities to B6 staged human blastocyst, as a structure composed of a monolayered cyst with an overall diameter of 150–250 μm comprising one inner cell cluster, and (2) on similarities to the molecular dynamic of human development as a structure that forms analogues of the three blastocyst cell lineages in the sequential and timely manner of a blastocyst. For example, >90% of morphologically adequate structures generated from the lines analysed formed >97% of analogues of three blastocyst-stage lineages (see Fig. 1h and Extended Data Fig. 3i). An exception is the line Shef6, which efficiently formed morphologically correct structures but appeared less efficient at forming PrE analogues. See also Supplementary Table 2. Blastoids reproducibly formed at high efficiency and we did not observe differences based on the number of passages after resetting in PXGL culture conditions. The effect of LPA, NAEPA (Sigma-Aldrich, N0912) and Verteporfin (Selleck Chemicals Llc, S1786) on the yield of blastoid formation was assessed by culturing naive PSC aggregates in PALY medium (without LPA) complemented with molecules added every day from 0 to 96 h. The Verteporfin treatment was executed without exposure to the light. The effect of the aPKC inhibitor CRT0103390 (a gift from the laboratory of K. Niakan) was assessed by culturing naive PSC aggregates in PALLY medium complemented with 2 µM CRT0103390 every day from 0 to 96 h. The formation of trophospheres was induced by culturing naive PSC aggregates in PALLY medium complemented with 2 µM XMU-MP-1 (Med Chem Express, HY-100526) or 3 µM SC-144 (Axon, 2324) every day from 0 to 96 h. The BSA concentration was titrated within the range of 0–0.3% for individual cell lines used for the formation of the blastoids and trophospheres. A step-by-step protocol is available on Protocol Exchange (10.21203/rs.3.pex-1639/v1).

### Derivation of cell lines from human blastoids

Derivation experiments were performed with blastoids cultured for 96 h as described in the previous section. Blastoids were individually transferred on gelatin-coated 96-well plates with feeder layers of gamma-irradiated MEFs. Naive PSCs were derived in PXGL medium^2^. TSCs were derived in human TSC medium^20^. After 24 h of culture on feeders, blastoids attached and, within one week, colonies were formed. Derivation was considered successful after three passages after blastoid transfer. For immunofluorescence assays, naive PSCs were transferred onto Geltrex (0.5 µl cm^−2^)-coated coverslips, and TSCs were transferred onto fibronectin-coated coverslips (5 μg ml^−1^, Sigma Aldrich, 08012).

### Trophoblast organoid formation

Organoid formation was performed with blastoid-derived TSC lines. Organoids were cultured as previously described^33^ with some modifications. Colonies of TSCs were dissociated into single cells using 1× trypsin at 37 °C for 5 min. After centrifugation, 200,000 cells were resuspended in 150 μl Matrigel (Corning, 356231). Droplets of 20 μl per well were placed into a prewarmed 48-well cell culture plate and placed upside down into the incubator for 20 min. Organoids were cultured in 250 ml TOM medium (Advanced DMEM-F12, N2 supplement, B27 supplement minus vitamin a, PenStrep, *N*-acetyl-l-cysteine (1.25 mM), l-glutamine (2 mM), A83-01 (500 nM), CHIR99021 (1.5 uM), recombinant human EGF (50 ng ml^−1^), 10% R-spondin 1 conditioned medium, recombinant human FGF2 (100 ng ml^−1^), recombinant human HGF (50 ng ml^−1^), PGE2 (2.5 μM). Medium was refreshed every other day. For SCT formation organoids were maintained in TOM medium until day 7.

### 2D trophoblast differentiations

The differentiation of blastoid derived TSCs was performed as described previously^20^ with some modifications. TSC lines were adapted to Fibronectin coating (5 μg ml^−1^, Sigma Aldrich, 08012) for at least three passages prior to the experiments. For EVT and SCT differentiation, cells were dissociated with TrypLE for 5 min at 37 °C and cells were seeded at a density of 55,000 cells per well onto 12-well plates. For SCT differentiation, the plates were precoated with 10 μg ml^−1^ fibronectin and cultured in SCT medium (DMEM/F12, supplemented with 0.1 mM 2-mercaptoethanol, 0.5% penicillin-streptomycin, 1% ITS-X supplement, 7.5 mM A83-01, 2.5 mM Y27632, 4% KnockOut Serum Replacement and 2 mM forskolin) for 3 days. For EVT differentiation, plates were precoated with Matrigel and cells were cultured in EVT medium (DMEM/F12, supplemented with 0.1 mM 2-mercaptoethanol, 0.5% penicillin-streptomycin, 1% ITS-X supplement, 2% Matrigel, 7.5 mM A83-01, 2.5 mM Y27632, 4% KnockOut Serum Replacement and 100 ng ml^−1^ NRG1). After three days, the medium was changed to EVT medium with 0.5% Matrigel and without NRG1. Cells were cultured until day 6.

### Human pre-implantation embryos culture

Human embryos were thawed following the manufacturer’s instructions (Cook Medical: Sydney IVF Thawing kit for slow freezing and Vitrolife: RapidWarmCleave or RapidWarmBlast for vitrification). Human embryos frozen at the 8-cell stage were loaded into a 12-well dish (Vitrolife: Embryoslide Ibidi) with non-sequential culture medium (Vitrolife G2 plus) under mineral oil (Origio: Liquid Paraffin) at 37 °C in 5% O_2_/6% CO_2_.

### Plasmid construction

The cDNA sequence of hYAP1, hYAP1(5SA) and hYAP1(5SA + S94A) were amplified from the pQCXIH-Myc-YAP, pQCXIH-Myc-YAP-5SA and pQCXIH-Myc-YAP-S94A plasmids, respectively. These YAP plasmids^34^ were gifts from K. Guan (Addgene #33091, #33093 and #33094). The individual cDNA sequences were cloned into pDONR211, followed by cloning into PB-TAC-ERP2 using Gateway (invitrogen) cloning strategy. PB-TAC-ERP2^35^ was a gift from K. Woltjen (Addgene plasmid #80478). Complete sequences of the resulting plasmids are available upon request.

### Cell transfection in human naive PSCs

pCAG-PBase (5 µg) and PB-TAC-YAP1-ERP (5 µg) were transfected by NEPA21 electroporation (Nepa Gene) into 5 × 10^4^ cells in single-cell suspension. Electroporated naive PSCs were plated on Geltrex (0.5 µl cm^−2^, Thermo Fisher Science, A1413302)-coated 6-well plates with PXGL medium containing Y-27632 (10 µM). Puromycin (0.5 μg ml^−1^, Sigma-aldrich, P7255) was added to PXGL medium from day 1 to day 3–4 to select transformed cells. pCAG-PBase was a gift from K. Woltjen.

### YAP overexpression in naive PSC aggregates

The naive PSC aggregates were formed from naive H9 cell lines integrated with the doxycycline inducible cassette as described in the section above. The aggregates were cultured in PALLY medium with reduced LPA concentration (5 nM) from 0 h to 48 h along with 100 ng ml^−1^ doxycycline. Higher LPA concentrations masked the effects of the genetic overexpression of the YAP1 variants. The number of cavitated aggregates was counted at 72 h.

### Single-cell RNA-seq library preparation and sequencing

To avoid over-representation of TE cells, blastoids were collected, dissociated and the cell suspension was stained using antibodies against TROP2 and PDGFRa that mark trophoblasts and primitive endoderm, respectively. For the 96 h time point, blastoids were selectively picked up from the microwell arrays before the dissociation, according to the morphological criteria described above. On the contrary, for the 24 and 65 h time points, all structures, including the ones that will not develop into a blastoid, were included. Accordingly, this non-selective picking correlated with the presence of more off-target cells. Cells were FACS-sorted into 384-well-plates containing the lysis buffer for Smart-seq2 and immediately frozen. The antibody staining was exploited in order to harvest specific numbers of TROP2^+^, PDGFRa^+^ and double-negative cells. The abutted FACS gates (DiVa 9.0.1) covered the whole spectrum and no blastoid cells were excluded. The H9 naive cells cultured on MEF were stained using an antibody against SUSD2, then FACS-sorted. Dead cells were excluded by DAPI staining. Smart-seq2 libraries were generated as described previously with minor optimization^36^. Maxima H Minus reverse transcriptase (3 U per reaction, Thermo Fisher Science, EP0751) was used for the cDNA synthesis. The prepared libraries were sequenced on the S1 or SP flow cell using an Illumina Novaseq instrument in 50-bp paired-end mode.

### Single-cell RNA-seq data analysis

Smart-Seq transcriptome sequencing experiments were analysed using genome sequence and gene annotation from Ensembl GRCh38 release 103 as reference. For gene-expression quantification RNA-seq reads were first trimmed using trim-galore v0.6.6 and thereafter aligned to the human genome using hisat2 v2.2.1. Uniquely mapping reads in genes were quantified using htseq-count v0.13.5 with parameter -s no. TPM estimates were obtained using RSEM v1.3.3 with parameter–single-cell-prior. Further analysis was performed in R v4.0.3 with Seurat v4.0.1. Based on initial evaluation of per-cell quality control metrics and outlier identification using the median absolute deviation algorithm, cells with ≤2,000 detected genes or ≥12.5% mitochondrial gene percentage were filtered out. Only genes detected in at least five cells were retained. Count-data were log-normalized, top 3,000 highly variable were selected, and standardization of per-gene expression values across cells was performed using NormalizeData, FindVariableFeatures and ScaleData data functions in Seurat. Principal component analysis (PCA) based on the standardized highly variable features was used for linear dimension reduction, a shared nearest neighbor (SNN) graph was constructed on the dimensionally reduced data, and the graph was partitioned using a SNN modularity optimization-based clustering algorithm at a range of resolutions using RunPCA, FindNeighbors and FindClusters from Seurat with default settings. Cluster marker and marker genes between identity groups were determined with the Wilcox likelihood-ratio test (two-sided) using the FindAllMarker and FindMarkers functions with *P*-value adjustment using Bonferroni correction and followed by filtering at a adjusted *P* value cut-off of 0.05. UMAP was used for visualization.

For integration of Smart-seq experiments from multiple sources we followed the previously described procedure^10^. Published data from E-MTAB-3929 (human preimplantation embryos^37^ ranging from embryonic day 3 to 7), GSE109555 (in vitro cultured blastocysts^38^) were downloaded, and data from Carnegie stage 7 embryo were kindly provided by the authors of the study^39^. All the data was preprocessed to obtain per gene read counts using the same protocol as described for blastoid cells, in the case of GSE109555 including adaptations to accommodate UMI and CB information following the authors’ instructions (https://github.com/WRui/Post_Implantation). For GSE109555 we used 1,000 cells randomly subsampled from the 3,184 high-quality single cells described in the original publication. Similar to ref. ^10^, we excluded cells belonging to haemogenic endothelial progenitors and erythroblasts. After evaluation of per-cell quality control metrics, and as in ref. ^10^, cells with >2,000 detected genes and <12.5% mitochondrial gene percentage were retained. Genes detected in at least five cells in any dataset were retained. log-normalization was performed using computeSumFactors in scran package v1.18.7, per-batch scaling normalization using multiBatchNorm in batchelor v1.6.3. Datasets were aligned using the fastMNN approach via SeuratWrappers v0.3.0 using the log-normalized batch-adjusted expression values. MNN low-dimensional coordinates were then used for clustering and visualization by UMAP. The data processing and analysis pipelines are publicly available at https://github.com/RivronLab/Human_Blastoid_Kagawa_et_al-.

### Bulk RNA-seq library preparation and sequencing

Bulk RNA-seq libraries were prepared using Smart-Seq2 protocol as previously described^36^. For each sample, 50 cells were pooled together and prepared for sequencing. The libraries were then sequenced using an Illumina Novaseq 6000 with 50-bp paired end mode. For each sample, approximately 10 million reads were obtained.

### Bulk RNA-seq data analysis

RNA-seq reads were first trimmed using trimgalore v0.5.0 and reads mapping to abundant sequences included in the iGenomes Ensembl GRCh38 bundle (rDNA, mitochondrial chromosome, phiX174 genome, adapter) were removed using bowtie2 v2.3.4.1 alignment. Remaining reads were analyzed using genome and gene annotation for the GRCh38/hg38 assembly obtained from Ensembl release 94. Reads were aligned to the genome using star v2.6.0c and reads in genes were counted with featureCounts (subread v1.6.2) and parameter -s 0. Differential gene-expression analysis on raw counts and variance-stabilized transformation of count data for heatmap visualization were performed using DESeq2 v1.18.1.

### Culture of human trophoblast stem cells and aggregate formation

Experiments were performed using the human blastocyst-derived TSC line bTS5 provided by the laboratory of T. Arima. Cells were cultured on Laminin 511 (5 µg ml^−1^, BioLamina, LN511) coated plates in TSC medium as previously described^20^. Aggregates of TSCs were formed as follows. Colonies were dissociated into single cells using Accutase at 37 °C for 5 min. The cells were resuspended into TSC medium containing 10 µM Y-27632, and 3.0 × 10^4^ cells were seeded onto a microwell array imprinted into a well of a 96-well plate. The same medium^20^ was refreshed daily. After 72 h, the aggregates were used for both characterization and implantation experiments.

### Endometrial organoid culture

Cryopreserved human endometrial organoids were provided by the H. Baharvand laboratory (Royan Institute) within the framework of collaboration agreements. Human endometrial organoids were established from healthy human donors following the protocol described previously^24,40^ with some modifications. In brief, organoids were cultured in human endometrial expansion medium composed of 10% R-spondin 1 conditioned medium (in-house made) and 10% noggin-Fc-conditioned medium^41^ (in-house made), supplemented with 1× N2 supplement, 1× B27 supplement, 1× insulin-transferrin-selenium (in-house), Glutamax (1 μM), *N*-acetylcysteine (1.25 mM, Sigma-Aldrich, A7250), nicotinamide (2.5 mM, Sigma-Aldrich, 72340), EGF (50 ng ml^−1^, Peprotech, 100-47), bFGF (2 ng ml^−1^, Peprotech, 100-18B), HGF (10 ng ml^−1^, Peprotech, 315-23), FGF10 (10 ng ml^−1^, Peprotech, 100-26), A83-01 (500 nM) and SB202190 (10 μM, Tocris, 1264). Y-27632 (10 μM) was used in the first 2 days after passaging to prevent apoptosis. The medium was changed every 2 days and the organoids were passaged with TrypLE followed by mechanical dissociation every 7–9 days.

### Hormonal stimulation of endometrial organoids and OFEL culture

Endometrial organoids were passaged as described in the previous section. The dissociated cells were resuspended in Matrigel supplemented with Y-27632 (10 µM), cell suspension was deposited in 48-well plates and were cultured in endometrial expansion medium for 2 days. The organoids were stimulated first with E2 (10 nM, Sigma-Aldrich, E2758) for 2 days, followed by the mixture of E2 (10 nM), P4 (1 μM, Sigma-Aldrich, P8783), and cAMP (250 μM, Biolog, B 007) with or without XAV939 (10 μM) (EPC or EPCX respectively) for 4 days. For OFEL culture, organoids were recovered from the matrigel droplets with ice-cold DMEM/F12 and mechanical pipetting. The organoids were dissociated using TrypLE and mechanically triturated to generate single cells and seeded at a density of 3–3.5 × 10^4^ cells per well into a 96-well glass bottom plate (Cellvis, P96-1.5H-N) and cultured for 2–3 days with stimulation. For contraceptive treatment, levonorgestrel^42^ (LNG) (10 μM, Sigma-Aldrich, PHR1850) was added every day to the medium after hormonal stimulation and continued until the end of the experiment.

### In vitro implantation assay

Confluent OFELs were prepared for the implantation assay at least 2 h prior to the deposition of blastoids, trophospheres, naive PSCs or TSCs aggregates by washing the OFEL two times with DMEM/F12 and adding mIVC1 medium^28^. Structures were then transferred onto the OFELs using a mouth pipette under an inverted microscope. After 24–48 h, the medium was removed, the well was washed with PBS, fixed using 4% formaldehyde for 30 min at room temperature and subsequently processed for immunofluorescence staining. The percentage of attached structures was reported as the percentage of total transferred structures.

### In vitro culture of human blastoids in post implantation conditions

Human blastoids were selected using a mouth pipette, washed with CMRL1066 medium and transferred into suspension culture plates or 96-well plates coated with Matrigel containing pre-equilibrated media adapted from monkey blastocyst culture^27^ with minor modifications as followed. For the first day, the culture medium was CMRL1066 supplemented with 10% (v/v) FBS, 1 mM l-glutamine (Gibco), 1× N2 supplement, 1× B27 supplement, 1 mM sodium pyruvate (Sigma) and 10 μM Y27632. After 24 h, half of the medium was replaced with a new medium including 5% Matrigel. After 48h, 50% of medium was replaced with a new medium supplemented with 20% (v/v) FBS and 5% Matrigel. After 72 h, half of the medium was replaced with a new medium supplemented with 30% (v/v) KSR and 5% Matrigel. Then, half of the medium was replaced every day and blastoids were cultured for up to 6 days. Cultures were fixed for staining after 4 and 6 days of in vitro culture with 4% PFA as mentioned above.

### Human pre-implantation embryos

The use of human embryos donated to research as surplus of IVF treatment was allowed by the French embryo research oversight committee: Agence de la Biomédecine, under approval numbers RE13-010 and RE18-010. All human pre-implantation embryos used in this study were obtained from and cultured at the Assisted Reproductive Technology unit of the University Hospital of Nantes, France, which is authorized to collect embryos for research under approval number AG110126AMP of the Agence de la Biomédecine. Embryos used were initially created in the context of an assisted reproductive cycle with a clear reproductive aim and then voluntarily donated for research once the patients had fulfilled their reproductive needs or the embryos had tested positive for the presence of monogenic diseases. Informed written consent was obtained from both parents of all couples that donated spare embryos following IVF treatment. Before giving consent, people donating embryos were provided with all of the necessary information about the research project and the opportunity to receive counselling. No financial inducements were offered for donation. Molecular analysis of the embryos was performed in compliance with the guidelines of the embryo research oversight committee and The International Society for Stem Cell Research (ISSCR)^43^.

### RNA extraction, cDNA synthesis and RT–qPCR

RNA was extracted using the RNeasy mini kit (Qiagen, 74106) and cDNA synthesis was performed using the Superscript III (Invitrogen, 18080093) enzyme. qPCR reactions were performed using GoTaq qPCR Master Mix (Promega, A6001) on CFX384 Touch Real-Time PCR Detection System (Bio-rad). Quantification was performed using Microsoft Office Excel by applying the comparative cycle threshold (*C*_t_) method. Relative expression levels were normalized to GAPDH. The primers used for the qPCR analysis are listed in Supplementary Table 3.

### ELISA assay for CGβ detection

Medium from wells containing unattached or attached blastoids was collected and centrifuged to remove debris and stored at −80 °C until use. The supernatant was subject to CGβ ELISA (Abcam, ab178633), according to the manufacturer’s instructions, alongside CGβ standards.

### Ligand–receptor analysis

The Cellinker web-platform was used to predict putative receptor–ligand interactions between polar TE and endometrial epithelial cells. Enriched genes in polar TE along with upregulated genes in stimulated OFELs were used as the query to search ligands and receptors in the database.

### Immunohistochemistry

The samples were fixed with 4% formaldehyde for 30 min at room temperature. Post fixation, formaldehyde solution was removed and the samples were washed at least three times with PBS. The samples were then permeabilized and blocked using 0.3% Triton X-100 and 10% normal donkey serum in PBS for at least 60 min. The samples were then incubated overnight at 4 °C with primary antibodies diluted in fresh blocking/permeabilization solution. The samples were washed with PBS containing 0.1% Triton X-100 (PBST) at least three times for 10 min each. The washing buffer was then replaced with Alexafluor tagged secondary antibodies (Abcam or Thermofisher scientific) along with a nuclear dye Hoechst-33342 (1:500 or 1:300 for 2D or 3D samples respectively, Life Technologies, H3570) diluted in PBST for 30 min in dark at room temperature. The samples were then washed with PBST three times for 10 min each. For human blastocysts, the samples were fixed at the B4 or B6 stage according to the grading system proposed by Gardner and Schoolcraft^44^ or at B3 or B4 +72 h in vitro culture. Embryos were fixed with 4% paraformaldehyde for 10 min at room temperature and washed in PBS/BSA. Embryos were permeabilized and blocked in PBS containing 0.2% Triton-x100 and 10% FBS at room temperature for 60 min. Samples were incubated with primary antibodies overnight at 4 °C. Incubation with secondary antibodies was performed for 2 h at room temperature along with Hoechst counterstaining. The samples were mounted for imaging in PBS in the wells of glass bottom micro slides (Ibidi, 81507). The details of antibodies and their dilutions along with stainings previously performed on human blastocysts (other studies) are provided in the Supplementary Tables 4, 5. EdU staining was done using Click-iT EdU Alexa Fluor 647 Imaging Kit (Thermo Scientific, C10640) following the manufacturer’s instructions.

### Microscopy and image analysis

The phase-contrast images were acquired using Thermo Fisher scientific EVOS cell imaging system and inverted wide-field microscope Axio VertA1. The number of blastoids or cavitated structures were counted manually for each well. After 96 h, a blastoid is defined based on the morphological parameters as described in previous sections. The fluorescent images and time-lapse images were acquired using Olympus IX83 microscope with Yokogawa W1 spinning disk (Software: CellSense 2.3; camera: Hamamatsu Orca Flash 4.0) or Nikon Eclipse Ti E inverted microscope, equipped with a Yokogawa W1 spinning disc (Software: Visiview 4.5.0.7 ; camera: Andor Ixon Ultra 888 EMCCD). The confocal images were analysed and display images were exported using FIJI 1.53k or Bitplane Imaris 9.7.0 softwares. For cell counting, Bitplane Imaris software was used. Cell count parameters were set for size and fluorescence strength of voxels and then overall cell count data was obtained for each image using the Imaris spot function. Note that large cavities in blastoids increase the depth of the imaging field and cause poor signal from deeply located cells. Therefore, our counting data in Figs. 1h, 3g could be underrepresented values, particularly in the case of trophectoderm cells. The quantification of the percentage of blastoids forming the NR2F2 axis was done manually. To do so, blastoids stained to detect NR2F2 expression were imaged using a confocal spinning disk microscope. The images were projected using a 3D-project function in FIJI. The blastoid was classified to have an axis when NR2F2 expression was restricted to its polar half with no expression or lower level of expression in the mural half. The inverted pattern of NR2F2 expression was classified as an invert axis. The blastoids with NR2F2 expression on their both polar and mural halves were classified to have no axis. Confocal immunofluorescence images of human blastocysts were acquired with a Nikon confocal microscope and a 20× mim or 25× silicon objective. Optical sections of 1 µm-thick were collected. The images were processed using Fiji (http://fiji.sc) and Volocity 6.3 visualization softwares. Volocity software was used to detect and count nuclei.

### Statistics and reproducibility

All the experiments were performed at least in three biological replicates unless specifically described in the Methods and the figure legends. Statistical analyses were performed using Graphpad prism 8.1.1 (330).

### Reporting summary

Further information on research design is available in the Nature Research Reporting Summary linked to this paper.

## Online content

Any methods, additional references, Nature Research reporting summaries, source data, extended data, supplementary information, acknowledgements, peer review information; details of author contributions and competing interests; and statements of data and code availability are available at 10.1038/s41586-021-04267-8.

## Supplementary information

Reporting Summary

Supplementary Table 1 Differentially expressed genes based on bulk and single-cell RNA-seq data. Tab1: Differentially expressed genes and enriched GO term are listed based on the comparison between PrE analogue cells isolated from blastoid and in vitro PrE stem cell culture (nEND and PrE cells: Linneberg-Agerholm et al., 2019). Tab2: Differentially expressed genes and enriched GO term are listed based on the comparison of TE analogue cells isolated from blastoid against in vitro TSCs in 2D (Okae et al., 2018), or TE analogue cells derived from trophosphere induced by XMU-MT-1 treatment and the comparison between TE analogue cells derived from trophosphere induced by SC-144 and naive PSC. Differentially expressed genes are listed based on the comparison of TE analogue cells isolated from blastoid against TSCs in 3D culture or TSC converted from naive PSC in 2D culture condition (Guo et al., 2021). Tab3: Differentially expressed genes and enriched GO term are listed based on the comparison of different cluster of cells in UMAP generated by single-cell RNA seq. The tables contain the comparison between cluster 4 (naive PSC) and cluster 10 (early TE analogue), cluster 2 (mural TE analogue) and cluster 10 (early TE analogue), cluster 2 (mural TE analogue) and cluster 5 (polar TE analogue), cluster 1 (EPI analogue) and cluster 6 (EPI analogue), cluster 6 (EPI analogue) and cluster 8 (PrE analogue), and cluster 4 (naive PSC) and cluster 0 (EPI analogue). Tab4: List of reported marker genes for EPI, TE and PrE are summarized.

Supplementary Table 2 Summary of the reported parameters and criteria for different protocols reporting blastocyst-like structures. This table includes reported (a) cell lines, (b) initial cell state, (c) molecules for the formation of blastocyst-like structures, (d) time of formation of blastocyst-like structures, (e) morphometric criteria used to define a blastocyst-like structure, (f) mean efficiency, (g) sequence of lineage specification, (h) transcriptomic state of the cells forming the blastocyst-like structures (quoted text comes from the corresponding paper). As a reference, the time of blastocyst formation and some of its morphometric criteria were also included.

Supplementary Table 3 A list of primers used for RT–qPCR.

Supplementary Table 4 A list of primary antibodies used for immunofluorescence staining.

Supplementary Table 5 Details of stainings previously performed on human blastocysts. This table includes reported (a) lineage, (b) marker gene, (c–h) reference information, (i) figure in this paper.

Video 1 Live imaging of forming blastoids. Live imaging of forming blastoids within microwells and then fixed, immunostained using GATA3 and OCT4 antibodies, and subsequently imaged.

Video 2 Full chip live imaging of forming blastoids. Live imaging of a full microwell chip with forming blastoids.

Video 3 Human blastoid attachment to a hormonally stimulated layer of endometrial cells. Live imaging of a human blastoid undergoing attachment to a hormonally stimulated layer of endometrial cells.

Video 4 Human blastoid polarly attached to a hormonally stimulated layer of endometrial cells. Imaging of two human blastoids attached via the polar region to a hormonally stimulated layer of endometrial cells, and challenged by pipetting liquid in their vicinity.

## Data Availability

Single-cell RNA-seq and bulk RNA-seq data for human blastoids used in this study were deposited at the Gene Expression Omnibus under the accession number GSE177689. Source data are provided with this paper.

## Source data

Source Data Fig. 1
Source Data Fig. 3
Source Data Fig. 4
Source Data Extended Data Fig. 1
Source Data Extended Data Fig. 3
Source Data Extended Data Fig. 5
Source Data Extended Data Fig. 6
Source Data Extended Data Fig. 7
Source Data Extended Data Fig. 8
Source Data Extended Data Fig. 9
Source Data Extended Data Fig. 10
