# Supplementary material for: Human blastoids model blastocyst development and implantation
Source: Nature. 2021 Dec 2;601(7894):600–5. doi: 10.1038/s41586-021-04267-8 (PMC8791832; doi:10.1038/s41586-021-04267-8)
Supplement: Supplementary file 1 — Reporting Summary [file 41586_2021_4267_MOESM1_ESM.pdf]

nature portfolio

Corresponding author(s): Nicolas Rivron

Last updated by author(s): Sep 14, 2021

Reporting Summary

Nature Portfolio wishes to improve the reproducibility of the work that we publish. This form provides structure for consistency and transparency in reporting. For further information on Nature Portfolio policies, see our Editorial Policies and the Editorial Policy Checklist.

Statistics

For all statistical analyses, confirm that the following items are present in the figure legend, table legend, main text, or Methods section.

n/a	Confirmed	
☐	☒	The exact sample size (*n*) for each experimental group/condition, given as a discrete number and unit of measurement
☐	☒	A statement on whether measurements were taken from distinct samples or whether the same sample was measured repeatedly
☐	☒	The statistical test(s) used AND whether they are one- or two-sided<br>*Only common tests should be described solely by name; describe more complex techniques in the Methods section.*
☐	☒	A description of all covariates tested
☒	☐	A description of any assumptions or corrections, such as tests of normality and adjustment for multiple comparisons
☐	☒	A full description of the statistical parameters including central tendency (e.g. means) or other basic estimates (e.g. regression coefficient) AND variation (e.g. standard deviation) or associated estimates of uncertainty (e.g. confidence intervals)
☐	☒	For null hypothesis testing, the test statistic (e.g. *F*, *t*, *r*) with confidence intervals, effect sizes, degrees of freedom and *P* value noted<br>*Give P values as exact values whenever suitable.*
☒	☐	For Bayesian analysis, information on the choice of priors and Markov chain Monte Carlo settings
☒	☐	For hierarchical and complex designs, identification of the appropriate level for tests and full reporting of outcomes
☒	☐	Estimates of effect sizes (e.g. Cohen's *d*, Pearson's *r*), indicating how they were calculated

Our web collection on statistics for biologists contains articles on many of the points above.

Software and code

Policy information about availability of computer code

Data collection	The phase contrast images of cultured cells or cell aggregates were acquired using Thermo Fisher scientific EVOS cell imaging system and inverted wide field microscope Axio VertA1.The fluorescent images and time-lapse images were acquired using Olympus IX83 microscope with Yokogawa W1 spinning disk (Software: CellSense 2.3 ; camera: Hamamatsu Orca Flash 4.0) or Nikon Eclipse Ti E inverted microscope, equipped with a Yokogawa W1 spinning disc (Software: Visiview 4.5.0.7 ; camera:Andor Ixon Ultra 888 EMCCD). Realtime PCR results were collected using CFX384 system (bio-rad). Single cell transcriptome libraries and bulk transcriptome libraries were sequenced using Illumina Novaseq 6000
Data analysis	The confocal images were analyzed using FIJI 1.53k, Bitplane Imaris 9.7.0 or Volocity 6.5 softwares.The cell-cell interactions were infered using the Cellinker Webpage (no version number available)FACS data were analysed using FACS DiVa 9.0.1All the statistical analysis were performed using Graphpad Prism 8.1.1 (330)Data was analyzed using trim-galore v0.6.6, hisat2 v2.2.1, htseq-count v0.13.5, RSEM v1.3.3, R v4.0.3, Seurat v4.0.1, computeSumFactors in scran package v1.18.7, multiBatchNorm in batchelor v1.6.3, SeuratWrappers v0.3.0.

For manuscripts utilizing custom algorithms or software that are central to the research but not yet described in published literature, software must be made available to editors and reviewers. We strongly encourage code deposition in a community repository (e.g. GitHub). See the Nature Portfolio guidelines for submitting code & software for further information.

Data

Policy information about availability of data

All manuscripts must include a data availability statement. This statement should provide the following information, where applicable:

- Accession codes, unique identifiers, or web links for publicly available datasets
- A description of any restrictions on data availability
- For clinical datasets or third party data, please ensure that the statement adheres to our policy

Raw data for single cell and bulk RNA sequencing data of blastoids were deposited at the GEO repository under the accession number GSE177689. Published human embryo data are available in the GSE109555, E-MTAB-3929.

Field-specific reporting

Please select the one below that is the best fit for your research. If you are not sure, read the appropriate sections before making your selection.

☒ Life sciences ☐ Behavioural & social sciences ☐ Ecological, evolutionary & environmental sciences

For a reference copy of the document with all sections, see nature.com/documents/nr-reporting-summary-flat.pdf

Life sciences study design

All studies must disclose on these points even when the disclosure is negative.

Sample size	No statistical methods were used to predetermine sample size. For each experiment, the sample size is determined based on an interval of confidence equal or above 95% as described in the methods and in the text, and on the sampling error, which is estimated based on the standard deviations during pilot studies.
Data exclusions	In single cell sequencing data, based on initial evaluation of per-cell quality control metrics and outlier identification using the median absolute deviation algorithm, cells with <= 2000 detected genes or >= 12.5% mitochondrial gene percentage were filtered out.
Replication	All attempts at replication were successful over 3 independent experiments.
Randomization	Samples were randomly allocated to groups prior to treatments which prevented any bias in the interpretation of data
Blinding	The investigators were blinded by preparing in advance the different cocktails and allocating numbers to the different treatments used to stimulate the stem cells.

Reporting for specific materials, systems and methods

We require information from authors about some types of materials, experimental systems and methods used in many studies. Here, indicate whether each material, system or method listed is relevant to your study. If you are not sure if a list item applies to your research, read the appropriate section before selecting a response.

Materials & experimental systems

n/a	Involved in the study	
☐	☒	Antibodies
☐	☒	Eukaryotic cell lines
☒	☐	Palaeontology and archaeology
☒	☐	Animals and other organisms
☐	☒	Human research participants
☒	☐	Clinical data
☒	☐	Dual use research of concern

Methods

n/a	Involved in the study	
☒	☐	ChIP-seq
☐	☒	Flow cytometry
☒	☐	MRI-based neuroimaging

Antibodies

Antibodies used

anti-NANOG (Abcam #ab109250, clone EPR2027(2), dilution 1:100)
anti-CDX2 (Emergo Europe #MU392A-5UC, clone CDX2-88, dilution 1:100)
anti-GATA4 (Invitrogen #14-9980-82, clone eBioEvan, dilution 1:400)
anti-OCT4 (Santacruz Biotechnology #sc-5279, clone C-10, dilution 1:100)
anti-GATA3 (Santacruz Biotechnology #sc-9009, clone H-48, dilution 1:100)
anti-GATA3 (Invitrogen #14-9966-82, clone TWAJ, dilution 1:200)
anti-ZO-1 (Invitrogen #339100, clone ZO1-1A12, dilution 1:100)
anti-CDH1 (eBioscience #14-3249-82, clone DECMA-1, dilution 1:250)

anti-aPKC (Santacruz Biotechnology #sc-216, clone MC5, dilution 1:100)
anti-CK7 (Abcam #ab181598, clone EPR17078, dilution 1:300)
anti-KLF17 (Sigma #HPA002926, clone NA (polyclonal), dilution 1:200)
anti-YAP1 (Santacruz Biotechnology #sc-101199, clone 63.7, dilution 1:100)
anti-NR2F2 (Santacruz Biotechnology #ab211776, clone EPR18442, dilution 1:100)
anti-AQP3 (ac on lines #ABIN863208, clone NA (ABIN863208), dilution 1:100)
anti-CGB (Dako #A0231, clone NA (Polyclonal), dilution 1:300)
anti-CGB (Abcam #ab9582, clone 5H4-E2, dilution 1:200
anti-PAEP (Abclonal #A5751, clone NA (Polyclonal), dilution 1:500)
anti-FOXA2 (Cell signal #8186, clone D56D6, dilution 1:1000)
anti-Acetylated Tubulin (Sigma #T7451, clone 6-11B-1, dilution 1:500)
anti-IFI16 (novusbio #NBP1-83118, clone NA (Polyclonal), dilution 1:100)
anti-GATA2 (Abcam #ab109241, clone EPR2822(2), dilution 1:250)
anti-TROP2 (R&D systems #MAB650, clone 77220, dilution 1:100 (IHC) 1:50 (FACS))
anti-PDGFRα (R&D systems #AF307, clone NA (Polyclonal), dilution 1:100 (IHC) 1:50 (FACS))
anti-SOX17 (R&D systems #AF1924-SP, clone EPR20684, dilution 1:200)
anti-KLF4 (Sigma #HPA002926, clone NA (Polyclonal), dilution 1:200)
anti-OTX2 (R&D systems #AF1979, clone NA (Polyclonal), dilution 1:100)
anti-SUSD2 (Miltenyibiotec #130-117-682, clone W5C5, dilution 1:100)
anti-CCR7 (Thermo scientific #MA5-31992, clone SR36-04, dilution 1:200)
anti-SOX2 (Invitrogen #14-9811-80, clone Btjce, dilution 1:200)
anti-MUC1 (Invitrogen #MA1-35039, clone 115D8, dilution 1:200)
anti-CD24 (BD Biosciences #561644, clone ML5, dilution 1:100)
anti-PODXL (R&D systems #MAB1658, clone 222328, dilution 1:100)
anti-TFAP2C (R&D systems #AF5059, clone NA (Polyclonal), dilution 1:100)

Validation

Validation statements available from manufacturers:
anti-NANOG (https://www.abcam.com/nanog-antibody-epr20272-ab109250.html)
anti-CDX2 (https://www.labome.com/product/Biogenex/MU392A-5UC.html)
anti-GATA4 (https://www.thermofisher.com/antibody/product/Gata-4-Antibody-clone-eBioEvan-Monoclonal/14-9980-82)
anti-OCT4 (https://www.scbt.com/p/oct-3-4-antibody-c-10_
anti-GATA3 (https://www.scbt.com/p/gata-3-antibody-h-48?productCanUrl=gata-3-antibody-h-48&_requestid=3381940)
anti-GATA3 (https://www.thermofisher.com/antibody/product/Gata-3-Antibody-clone-TWAJ-Monoclonal/14-9966-82)
anti-ZO-1 (https://www.thermofisher.com/antibody/product/ZO-1-Antibody-clone-ZO1-1A12-Monoclonal/33-9100)
anti-CDH1 (https://www.thermofisher.com/antibody/product/CD324-E-Cadherin-Antibody-clone-DECMA-1-Monoclonal/14-3249-82)
anti-aPKC (https://www.scbt.com/p/pkc-zeta-antibody-c-20)
anti-CK7 (https://www.abcam.com/cytokeratin-7-antibody-epr17078-cytoskeleton-marker-ab181598.html)
anti-KLF17 (https://www.sigmaaldrich.com/AT/en/product/sigma/hpa002926)
anti-YAP1 (https://www.scbt.com/p/yap-antibody-63-7)
anti-NR2F2 (https://www.abcam.com/nr2f2-antibody-epr18442-ab211776.html)
anti-AQP3 (https://www.antibodies-online.com/antibody/863208/anti-Aquaporin+3+Gill+Blood+Group+AQP3+C-Term+antibody/)
anti-CGB (https://www.labome.com/product/Dako/A0231.html)
anti-CGB (https://www.abcam.com/hcg-beta-antibody-5h4-e2-ab9582.html)
anti-PAEP (https://abclonal.com/catalog-antibodies/PAEPPolyclonalAntibody/A5751)
anti-FOXA2 (https://www.cellsignal.at/products/primary-antibodies/foxa2-hnf3b-d56d6-xp-rabbit-mab/8186)
anti-Acetylated Tubulin (https://www.sigmaaldrich.com/AT/en/product/sigma/t7451?
gclid=Cj0KCQjw5uWGBhCTARIsAL70sLKfFm6GzCha4llea9SnzsZ5NvDKPNukmZNa61-V6xRBwJWw0GbCU6gaAmeSEALw_wcB)
anti-IFI16 (https://www.novusbio.com/products/ifi16-antibody_nbp1-83118)
anti-GATA2 (https://www.abcam.com/gata2-antibody-epr28222-ab109241.html)
anti-TROP2 (https://www.rndsystems.com/products/human-trop-2-antibody-77220_mab650)
anti-PDGFRα (https://www.rndsystems.com/products/human-pdgf-ralpha-antibody_af-307-na)
anti-SOX17 (https://www.rndsystems.com/products/human-sox17-antibody_af1924)
anti-KLF4 (https://www.sigmaaldrich.com/AT/en/product/sigma/hpa002926)
anti-OTX2 (https://www.rndsystems.com/products/human-otx2-antibody_af1979)
anti-SUSD2 (https://www.miltenyibiotec.com/AT-en/products/susd2-antibody-anti-human-w5c5.html#gref)
anti-CCR7 (https://www.thermofisher.com/antibody/product/CCR7-Antibody-clone-SR36-04-Recombinant-Monoclonal/MA5-31992)
anti-SOX2 (https://www.thermofisher.com/antibody/product/SOX2-Antibody-clone-Btjce-Monoclonal/14-9811-82)
anti-MUC1 (https://www.thermofisher.com/antibody/product/MUC1-Antibody-clone-115D8-Monoclonal/MA1-35039)
anti-CD24 (https://www.bdbiosciences.com/en-us/products/reagents/flow-cytometry-reagents/research-reagents/single-color-antibodies-ruo/alexa-fluor-647-mouse-anti-human-cd24.561644)
anti-PODXL (https://www.rndsystems.com/products/human-podocalyxin-antibody-222328_mab1658)
anti-TFAP2C (https://www.rndsystems.com/products/human-ap-2gamma-antibody_af5059)

Eukaryotic cell lines

Policy information about cell lines

Cell line source(s)

embryonic stem cell lines: Shef6, HNES1 and iPSC lines: cR-nCRM2 and niPSC16.2.b were provided by the laboratory of Austin Smith
H9 and H9-GFP reset to naive state were provided by the Laboratory of Yasuhiro Takashima
hTSC line bTS5 was provided by the laboratory of Takahiro Arima
Endometrial organoids were provided by the laboratory of Hossein Baharvand

Authentication

H9 (primed and naive PSCs) and TSC-bTS5 were included in single cell sequencing analysis to authenticate their identity

Mycoplasma contamination	Cells were routinely tested for mycoplasma contamination. No contamination was detected.
Commonly misidentified lines (See ICLAC register)	no misidentified lines were used in the study

Human research participants

Policy information about studies involving human research participants

Population characteristics	Not applicable because no human subjects were involved in this research. We used donated embryos surplus to IVF treatment and endometrial organoids from a biobank.
Recruitment	Informed consent was obtained from all couples that donated spare embryos following IVF treatment and from people that previously donated endometrial samples that constituted the endometrial biobank used in this study.
Ethics oversight	The use of human embryos donated to research as surplus of IVF treatment was allowed by the French embryo research oversight committee: Agence de la Biomédecine, under approval number RE13-010 and RE18-010. All human pre-implantation embryos used in this study were obtained from and cultured at the Assisted Reproductive Technology unit of the University Hospital of Nantes, France, which are authorized to collect embryos for research under approval number AG110126AMP of the Agence de la Biomédecine. Human endometrium samples were obtained from patients who signed an informed consent form and protocols approved by the Ethics Committee of Royan Institute (IR.ACECR.ROYAN.REC. 1397.93) and ethical approval from the Ethics Committee of the Shahid Beheshti University of Medical Sciences (IR.SBMU.MSP.REC. 1396.25). The Wicell line H9 was used under the agreement 20-WO-341 for a research program entitled 'Modeling early human development: Establishing a stem cell based 3D in vitro model of human blastocyst (blastoids)'. Blastoid generation was approved by the Commission for Science Ethics of the Austrian Academy of Sciences. All experiments complied with all relevant guidelines and regulations, including the 2021 ISSCR guidelines that forbid the transfer of human blastoids into an uterus.

Note that full information on the approval of the study protocol must also be provided in the manuscript.

Flow Cytometry

Plots

Confirm that:

☒ The axis labels state the marker and fluorochrome used (e.g. CD4-FITC).

☒ The axis scales are clearly visible. Include numbers along axes only for bottom left plot of group (a 'group' is an analysis of identical markers).

☒ All plots are contour plots with outliers or pseudocolor plots.

☒ A numerical value for number of cells or percentage (with statistics) is provided.

Methodology

Sample preparation	Cells were collected by dissociated blastoids with sequential treatment of 300units/ml collagenase IV for 30minutes and 0.5% Trypsin (10x) for 20minutes. Cells were stained with Trop2 and PDGFRa antibodies followed by the secondary antibodies
Instrument	FACS Aria III (BD)
Software	DiVa version on the F02 is 9.0.1.
Cell population abundance	Abundance of the cell populations of interest was determined by the appropriate negative control and the purity of sorted population was assessed by the post sort analysis.
Gating strategy	FSC-A/SSC-A and SSC-H/SSC-W gates were applied to remove debris, and non-single cell aggregates respectively. Dead cells were excluded by using DAPI signal. A example of FACS gating strategy is available at the Supplementary Figure 2A.

☒ Tick this box to confirm that a figure exemplifying the gating strategy is provided in the Supplementary Information.
